# Multiparametric Analyses of Human PBMCs Loaded *Ex Vivo* with a Candidate Idiotype Vaccine for HCV-Related Lymphoproliferative Disorders

**DOI:** 10.1371/journal.pone.0044870

**Published:** 2012-09-18

**Authors:** Annacarmen Petrizzo, Maria Lina Tornesello, Maria Napolitano, Giovanna D'Alessio, Angelo Salomone Megna, Riccardo Dolcetti, Valli De Re, Ena Wang, Franco M. Marincola, Franco M. Buonaguro, Luigi Buonaguro

**Affiliations:** 1 Laboratory of Molecular Biology and Viral Oncogenesis, National Cancer Institute “Fond. G. Pascale”, Naples, Italy; 2 Laboratory of Clinical Immunology, National Cancer Institute “Fond. G. Pascale”, Naples, Italy; 3 Division of Infectious Diseases, Rummo Hospital, Benevento, Italy; 4 Cancer Bio-Immunotherapy Unit, Centro di Riferimento Oncologico, National Cancer Institute, Aviano, Italy; 5 Experimental and Clinical Pharmacology, Centro di Riferimento Oncologico, National Cancer Institute, Aviano, Italy; 6 Infectious Disease and Immunogenetics Section (IDIS), Department of Transfusion Medicine, Clinical Center, and trans-NIH Center for Human Immunology, National Institutes of Health, Bethesda, Maryland, United States of America; St.Louis University, United States of America

## Abstract

Hepatitis C virus (HCV) has been identified as one of the major risk factors for type II mixed cryoglobulinemia (MC), during the clinical evolution of chronic hepatitis, which may lead to development of B cell non-Hodgkin's lymphoma (NHL). We have previously shown that the candidate idiotype vaccine, based on the IGKV3-20 light chain protein, is able to induce activation and maturation of circulating antigen presenting cells (APCs) in both HCV-positive and HCV-negative healthy control subjects, with production of Th2-type cytokines. Here, the effect of the recombinant IGKV3-20 protein on human peripheral blood mononuclear cells (PBMCs) from HCV-positive subjects, with known blood levels of cryoglobulins, is shown via gene expression profiling analysis combined to multiparameter flow cytometry and multiplex analyses of cytokines.

## Introduction

Hepatitis C virus (HCV) is a Hepacivirus of the Flaviviridae family, mainly involved in hepatic disorders, including chronic hepatitis, cirrhosis and hepatocellular carcinoma (HCC) [1].

HCV has also been implicated as one of the major risk factors for type II mixed cryoglobulinemia (MC), an autoimmune disease leading to B cell non-Hodgkin's lymphoma (NHL) in about 10% of MC patients [2], [3], [4], [5].

The most accredited pathogenetic mechanism of MC during HCV chronic infection is the persistent immune stimulation of B-cell compartment by viral proteins (e.g. HCV E2 protein) which drives the expansion of cellular clones ultimately resulting in production of cross-reactive autoantibodies, including cryoglobulins [6], [7]. Further chromosomal aberrations, inducing elevated expression of Bcl-2, lead to inhibition of apoptosis and increased B-cell survival [8] which may evolve into a B cell NHL as late complication of the MC syndrome [9], [10].

Clonal B-lymphocyte expansion is characterized by the production of an immunoglobulin presenting a unique antigen-specific region, named idiotype (Id). Therefore, the Id can be a suitable target for active and passive immune-therapeutic strategies to eliminate clonal B cells driving the tumor [11]. However, each individual patient needs to be characterized, in order to identify the autologous Id expressed by the clonal tumor B cells and to develop the patient-specific vaccine.

Several clinical trials, up to Phase III, have been performed to evaluate safety and efficacy of autologous idiotype vaccines targeting tumors of hematological origin (http://clinicaltrials.gov/ct2/results?term=idiotypevaccine&pg=1). In particular, one of the Phase III efficacy trials has shown a prolonged disease-free survival (DFS) in follicular lymphoma patients vaccinated with patient-specific hybridoma-derived Id vaccine after chemotherapy-induced complete response (CR) or CR unconfirmed (CRu) [12]. Additional clinical trials with autologous idiotype vaccines have proven to be effective in inducing specific immune responses, which are able to kill *in vivo* residual follicular lymphoma cells after chemotherapy [13], [14], ultimately resulting in prolonged survival of responding patients [15].

Three clinical trials evaluating autologous idiotype vaccines are currently recruiting volunteers. A Phase I clinical trial aims to assess the safety of an autologous recombinant idiotype vaccine for the treatment of patients with relapsed or transformed follicular lymphoma (ClinicalTrials.gov Identifier: NCT01022255).

Furthermore, a Phase II clinical trial is testing the efficacy of a specific autologous idiotype vaccine in preventing relapse of follicular lymphoma. The vaccine is conjugated to keyhole limpet hemocyanin (KLH) and granulocyte macrophage colony stimulating factor (GM-CSF) (ClinicalTrials.gov Identifier: NCT00530140).

Finally, a randomized Phase II clinical trial is aiming to evaluate the outcome of a combination strategy based on infusion of CD3/CD28 autologous T cells, primed ex vivo with Id-KLH, and vaccine administration in patients with multiple myeloma (ClinicalTrials.gov Identifier: NCT01426828).

However, the need of patient-tailored autologous Id vaccines represents a major limitation for their large-scale implementation and the search for shared idiotypes among patients with lymphoproliferative disorders is actively pursued by different groups [16], [17], [18], [19], [20], [21]. This would allow, indeed, the use of limited number of Ids for vaccine development.

In this respect, clonal B cells involved in HCV-associated type II MC as well as in NHL from different patients carry closely homologous (“stereotyped”) complementarity-determining region 3 (CDR3) sequences. In particular, the IGHV1–69, IGHV3–7, IGHV4–59 variable heavy (VH)- and IGKV3–20 and IGKV3–15 variable light (VL)-chain genes are the most represented [20], [22], [23], suggesting a model of antigen-driven origin for such lymphoproliferative disorders with the recognition of a limited subset of HCV antigens [24], [23].

The IGKV3-20 idiotype has been selected as potential target of either passive immune therapy or active vaccine strategy. Highly reactive monoclonal antibodies (MAbs) specific for IGKV3-20 idiotype of a subject with HCV infection and type II MC associated NHL have been generated, showing their reactivity with B-cell receptors (BCRs) of lymphoma cells from different patients [21]. Alternatively, the IGKV3-20 idiotype has been proposed as vaccine molecule in order to elicit a humoral/cellular immune response as preventive and/or therapeutic approach against the B cell clone sustaining the HCV-associated NHL [25].

We have previously reported a multiparametric analysis of the effects induced in human PBMCs after *ex vivo* exposure to the IGKV3-20 idiotype protein [26]. This strategy may represent, indeed, a reliable surrogate for testing immunogenicity of vaccine products and provide relevant information for optimization through the process of vaccine development.

The results showed that IGKV3-20-induced expression of activation markers and co-stimulatory molecules in the evaluated circulating antigen presenting cells (APCs), CD14^+^ monocyte as well as CD123^+^ plasmacytoid DC (pDC) or CD11c^+^ myeloid DC (mDC) populations, is largely comparable between HCV-positive and control subjects. No significant difference was observed between results obtained in human monocyte-derived dendritic cells (MDDCs) and circulating APCs, confirming previous results from us and other groups [27], [28], [29], [30], [31]. This observation suggests that the HCV positivity status does not significantly impair the immune activation status and the responsiveness of circulating APC populations to the IGKV3-20 immunogenic stimulus. Nonetheless, some HCV-positive individuals show a complete lack of maturation induced by IGKV3-20 in circulating APCs, strongly suggesting the need for individual evaluations to identify possible impairments in response to this immunogen. Moreover, IGKV3-20 induced a significantly higher production of Th2 cytokines in PBMCs from HCV-positive and control subjects, compared to Th1 cytokines. However, the levels of Th2 cytokines induced in the HCV-positive samples were significantly higher than those identified in the control samples (*p*<0.01), suggesting the persistence of a prevalent Th2 status [26].

In the present study, the effect of the recombinant IGKV3-20 protein on human peripheral blood mononuclear cells (PBMCs) from HCV-positive subjects, with known blood levels of cryoglobulins, is shown via gene expression profiling analysis combined to multiparameter flow cytometry and multiplex analyses of cytokines.

## Materials and Methods

### Enrolled subjects

Peripheral blood was obtained by venipuncture from 5 HCV-negative healthy control volunteers and 10 HCV-positive subjects. All human specimens were obtained at the Infectious Disease Unit of “G. Rummo” Hospital (Benevento) under informed consent, as approved by the Institutional Review Board of the “Rummo” Hospital, and processed at the National Cancer Institute in Naples.

### PBMC isolation

Fresh human peripheral blood mononuclear cells (PBMCs) were isolated by Ficoll-Hypaque density gradient centrifugation and plated in six-well plates at a concentration of approximately 1×10^7^ cells/well in a maximum volume of 3 ml/well [32], [33]. Isolated PBMCs were incubated for 24 h (short-term culture) or for 6 days (medium-term culture) in RPMI 1640 medium.

### Cell culture medium

PBMCs culture medium consisted of RPMI 1640 (Life Technologies, Carlsbad, CA) supplemented with 2 mM L-glutamine (Sigma), 10% fetal calf serum (Life Technologies) and 2% penicillin/streptomycin (5,000 I.U./5 mg per ml, MP Biomedicals). Recombinant interleukin-2 (rIL-2; R&D Systems, Minneapolis, Minn.) was added at a concentration of 75 U/ml for medium-term culture (6 days).

### Cell treatment

PBMCs were incubated for 24 hours (short-term culture) with the recombinant IGKV3-20 protein (15 µg/ml, endotoxin-free) provided by Areta International (Gerenzano, Italy). In parallel, cells were pulsed with 8 µg/ml of lipopolysaccharide (LPS), as positive control. Alternatively, PBMCs were incubated for 6 days (medium-term culture) with the same concentration of recombinant IGKV3-20 protein or LPS, added at day 0 and 3. PBS was used as negative control. At the end of the incubation, PBMCs were harvested, washed with 1× PBS (137 mM NaCl, 2.7 mM KCl, 10 mM Na_2_HPO_4_, 2 mM KH_2_PO_4_, pH 7.2) without Calcium and Magnesium and analyzed by flow cytometry or stored frozen at −80°C in RNA later (Ambion, Austin, TX).

All cell supernatants were collected for quantification of cytokine production by enzyme-linked immunosorbent assay (ELISA).

### Flow cytometry

Short-term culture PBMCs were incubated for 30 min at 4°C with human monoclonal antibodies specific for CD3, CD40, CD80, CD83, CD86, HLA-DR, CD11c and CD14 (BD Pharmingen, San Diego, CA), washed and then analysed with a FACScalibur flow cytometer (BD Pharmingen). Data analysis was carried out with WinMDI2.8 Software. A Paired t test was performed, all p-values were two-tailed and considered significant if less than 0.05.

### Cytokine analysis

At the time of cell harvesting, supernatants were collected and analyzed. Cytokine production was assessed using the Instant ELISA system (Bender Medsystems) for quantitative detection of human cytokines, according to the manufacturer's instructions. Data acquisition was performed using the Sirio-S ELISA reader. A Paired t test was performed, all p-values were two-tailed and considered significant if less than 0.05.

### RNA purification and microarray hybridization

Total RNA from PBMCs was purified using the RNeasy Mini Kit (Qiagen) according to manufacturer's instructions. The purity of total RNA preparation was verified by 260∶280 nm ratio (range, 1.8–2.0), at NanoDrop spectrophotometer (Thermo Fisher Scientific, Waltham, MA). In addition, phenol contamination was checked and a 2.0–2.2 ratio at 260∶230 nm was considered acceptable. Integrity of extracted RNA was evaluated using the LabChip GX/GXII Electrophoresis System (Caliper LifeSciences) and a RNA Quality Score (RQS) >8 was considered acceptable for RNA quality.

Single-stranded cDNA was obtained with the Ambion WT Expression Kit (Applied Biosystems) according to manufacturer's instructions from a starting material of 200 ng total RNA per sample. The sense strand cDNA was fragmented and labeled using the Affymetrix GeneChip WT Terminal Labeling Kit (Applied Biosystems) according to manufacturer's instructions.

After hybridization on Human Gene 1.0 ST Arrays (Affymetrix) for 16 h at 45°C at 60 rpm in a Hybridization Oven 640 (Affymetrix), slides were washed and stained on a Fluidics Station 450 (Affymetrix). Scanning was performed on a GeneChip Scanner 3000 7G (Affymetrix) and Affymetrix GCOS software was used to perform image analysis and generate raw intensity data (CEL files). Initial data quality was assessed by background level and pair-wise correlation among samples. A log_2_ base transformation was applied to the data before the arrays were normalized. Microarray intensity data of probe sets were normalized by RMA, which includes global background adjustment and quantile normalization.

### Unsupervised Analysis

For the unsupervised analysis a low-stringency filtering was applied, selecting the genes differentially expressed in 80% of all experiments with a >3 fold change ratio in at least one experiment. Hierarchical cluster analysis was conducted on the selected genes according to Eisen et al. [34]; differentially expressed genes were visualized by Treeview and displayed according to the central method [35], [34].

### Supervised Analysis

Supervised class comparison was performed using BRB ArrayTool developed at NCI, Biometric Research Branch, Division of Cancer Treatment and Diagnosis. Two subsets of genes were explored. The first subset included genes up-regulated in stimulated (IGKV3-20 treated) PBMCs compared to non-stimulated (PBS treated) PBMCs after 24 h incubation; the second subset included genes up-regulated in stimulated PBMCs compared to non-stimulated PBMCs after 6 days incubation from both HCV-positive subjects and healthy controls.

Class comparison analyses were tested for an univariate significance threshold set at a *p*-value<0.001. Gene clusters identified by the univariate *t*-test were challenged with two alternative additional tests, an univariate permutation test (PT) and a global multivariate PT.

Class comparison and hierarchical clustering were employed to determine the pattern of response and results are illustrated as a heat map of significance values. All analyses were performed using R and Cytoscape (http://www.cytoscape.org); gene function was assigned based on Database for Annotation, Visualization and Integrated Discovery (DAVID) (http://www.david.abcc.ncifcrf.gov) and Gene Ontology (http://www.geneontology.org).

### Ingenuity Pathways Analysis

Ingenuity Pathways Analysis (IPA, www.ingenuity.com) was employed to elucidate the relationship and connection between differentially expressed genes.

The IPA system transforms large data sets into a group of relevant networks containing direct and indirect relationships between genes, based on interactions contained in the Ingenuity Pathways Knowledge Base. An IPA “network” is a graphical representation of the molecular relationships between genes, represented as nodes, and biological interactions, represented as connecting lines between nodes. Gene networks are generated algorithmically based on connectivity in terms of expression, activation, transcription and/or inhibition.

## Results

### Clinical parameters of subjects included in the analysis

Fifteen subjects were enrolled for the present study. Ten subjects were HCV-positive, of whom six were males and four were females. Five healthy donors were enrolled as controls, matched for age and life style. Clinical parameters of enrolled subjects are described in Table 1.

**Table 1:** Clinical parameters of enrolled subjects.

SAMPLE ID	SEX	HCV RNA (IU/ml)	CRYOCRIT %	RF TEST (IU/ml)	WAALER-ROSE TEST
S.R.	F	NEG.	NEG.	NEG.	NEG.
A.I.	F	NEG.	NEG.	NEG.	NEG.
S.F.	M	NEG.	NEG.	NEG.	NEG.
C.R.	F	NEG.	NEG.	NEG.	NEG.
A.C.	F	NEG.	NEG.	NEG.	NEG.
N.L.	M	1,900	1.50	31.2	POS.
M.M.L.	F	629,000	0.5	127	POS.
F.M.	F	500,000	0.1	3.9	NEG.
P.M.	F	700,000	2	607	POS.
V.A.	M	3,000,000	0.5	7.7	NEG.
B.E.	M	9,000	1.1	670	POS.
D.N.	M	1,130,000	0.3	122.00	POS.
L.M.R.	M	5,850,000	0.8	114	POS.
D.B.A.	M	2,340,000	0.6	112	POS.
B.D.	F	NEG. (anti HCV+)	0.2	12	NEG.

### Maturation markers induced in circulating APCs by the recombinant IGKV3-20 protein

In order to evaluate the maturation markers induced by recombinant IGKV3-20 protein, the expression of CD40, CD80, CD83, CD86 and HLA-DR was examined in circulating APCs (i.e. CD14^+^ monocytes, and CD11c^+^ myeloid dendritic cells - mDCs) by flow cytometry.

To this aim, both monocytes as well as mDCs were identified as positive for HLA-DR and negative for CD3 (DR^+^/CD3^−^). Such selected cells were further gated into CD14^+^ (monocytes) or CD14^−^/CD11c^+^ (mDCs).

**Figure 1:**
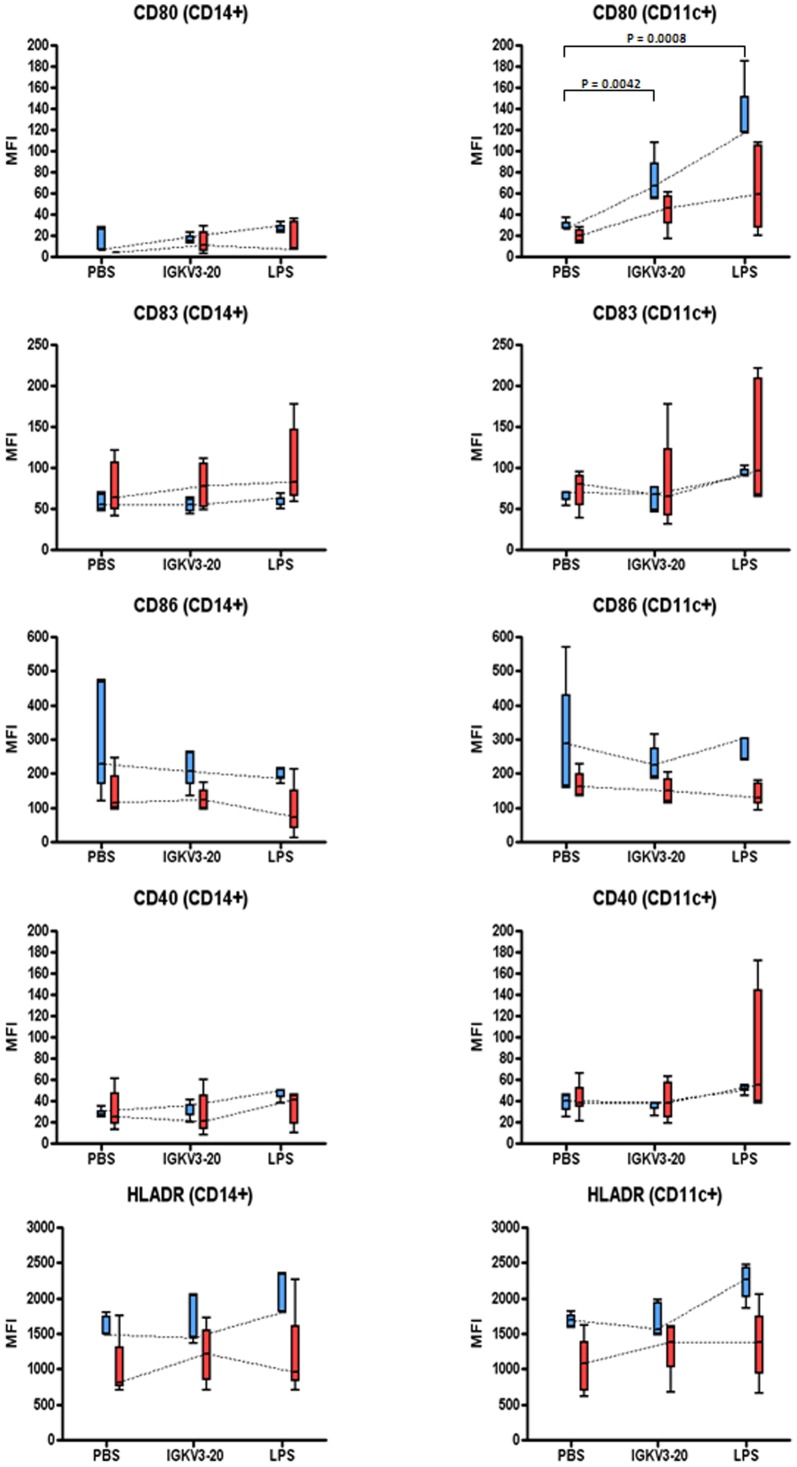
Expression of surface maturation/activation markers in PBMCs. Mean Fluorescence Index (MFI) for each of the indicated markers was evaluated on cells after 24 h treatment with IGKV3-20 and LPS. Mean MFI values in each group are linked by dotted line. HCV-negative samples = blue; HCV-positive samples = red. P-value is indicated only when <0.01.

The basal expression of all the evaluated markers was largely comparable between HCV-negative and positive subjects with no statistically significant differences, although a trend of higher expression in both CD14^+^ and CD11c^+^ cell populations from healthy controls was observed for all markers (Fig. 1). The only exception was represented by basal CD83 expression, which showed a trend of higher expression in cells from HCV-positive subjects (Fig. 1).

On the contrary, the stimulation with recombinant IGKV3-20 protein induced a trend of increased expression of the activation/maturation markers in both CD14^+^ and CD11c^+^ cell populations from HCV-positive subjects and healthy controls. In particular, the observed increased expression was due to a higher number of surface markers per cell rather than an increase in the absolute number of CD14^+^ and CD11c^+^ cells expressing such markers.

Of note, a statistically significant up-regulation of CD80 expression was observed in CD11c^+^ cell populations from the HCV-negative subjects (p-value 0.0042) (Fig. 1).

### Cytokine pattern induced in circulating APCs by the recombinant IGKV3-20 protein

**Figure 2:**
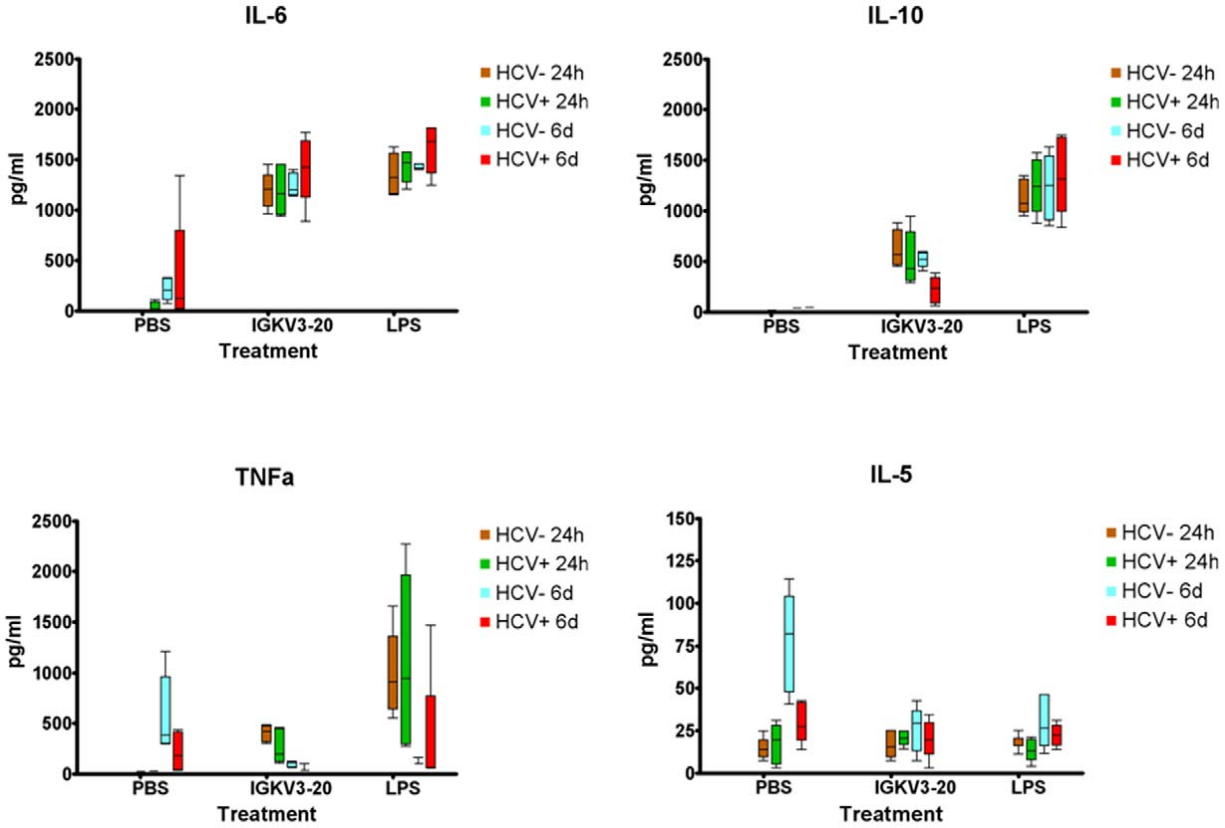
Analysis of cytokine production in supernatants of PBMCs. Production of indicated cytokines was evaluated in supernatants of PBMCs from HCV-negative and positive subjects, induced by IGKV3-20 and LPS after 24 h and 6 d incubation.

The level of Th1 (IL-2 and TNF-α) and Th2 (IL-5, IL-6, IL-10) cytokines was assessed by ELISA in supernatants of PBMCs, after 24 h or 6 days of incubation with IGKV3-20 (Fig. 2).

The results show that IGKV3-20 protein induces a significant production of both Th1 and Th2 cytokines (p-value<0.01) with a prevalent Th2-biased cytokine pattern, in both HCV-negative and positive subjects. In particular, the increased production of the Th2 IL-6 and IL-10 persists for the 6 days of induction, whereas the induction of the Th1 TNF-α fades away at 6 days (Fig. 2). On the contrary, both IL-5 and IL-2 are not induced by IGKV3-20 (Fig. 2 and data not shown).

In particular, the induction of the anti-inflammatory IL-10 suggests the activation of a positive homeostatic mechanism to counteract an excessive pro-inflammatory condition which would be detrimental to an effective immune response, as previously reported by our group [26].

### Unsupervised analysis

The gene expression profile of samples from 4 healthy controls and 5 HCV-positive subjects was analyzed using the Human Gene 1.0 ST Arrays, which interrogates 28,869 well-annotated human genes (Affymetrix).

Total RNA was extracted from PBMCs and its quality was verified with the LabChip GX/GXII Electrophoresis System. Discrete 28S and 18S ribosomal RNA bands, as well as a 28S/18S area ratio close to 2 were the two parameters taken into consideration for subsequent gene expression profile analysis.

Based on this selection strategy, all control and HCV-positive samples from non-stimulated (PBS) and stimulated (IGKV3-20 or LPS) PBMCs, after 24 h or 6 days incubation, were included and compared by unsupervised analysis.

According to filtering parameters described in Materials and Methods section, 6,562 differentially expressed genes were selected for the unsupervised analysis.

**Figure 3:**
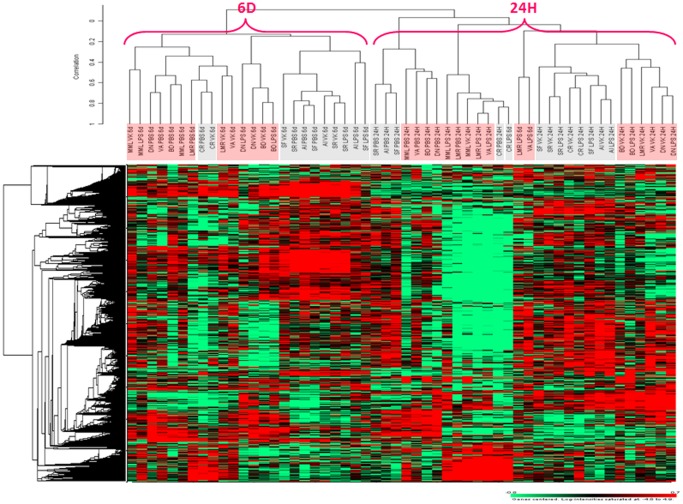
Unsupervised hierarchical clustering. Heat map including all samples from 4 healthy controls (light blue) and 5 HCV-positive subjects (light red). Red indicates over-expression; green indicates under-expression; black indicates unchanged expression; gray indicates no detection of expression. Each row represents a single gene; each column represents a single sample. The dendrogram at the left of matrix indicates the degree of similarity among the genes examined by expression patterns. The dendrogram at the top of the matrix indicates the degree of similarity between samples.

A clear cut separation of samples in two main clusters according to treatment duration (24 h vs 6 days) is observed, regardless the cell treatment (Fig. 3). The only exception is represented by two HCV-positive samples, treated with LPS for 6 days, which fall into the 24 h-cluster. Moreover, within the 24 h-cluster a highly significant sub-clustering of all non-stimulated samples (PBS) is observed, except for a single sample, with a distinct separation according to the HCV positivity status. On the contrary, samples stimulated with IGKV3-20 or LPS cluster together and their separation is generally based on the HCV positivity status and, in some cases, appears to be more related to the specific responsiveness of the different subjects to both stimulations (Fig. 3).

On the other hand, in the 6 d-cluster such clear sub-clustering of non-stimulated samples is not observed, and 3 of 9 samples cluster with stimulated samples. Moreover, samples treated with IGKV3-20 or LPS show a similar pattern of clustering as in the 24 h-cluster, generally based upon the HCV positivity status and, in some cases, related to the specific responsiveness of the different subjects to both treatments (Fig. 3).

### Genes transcriptionally activated by IGKV3-20 in PBMCs

**Figure 4:**
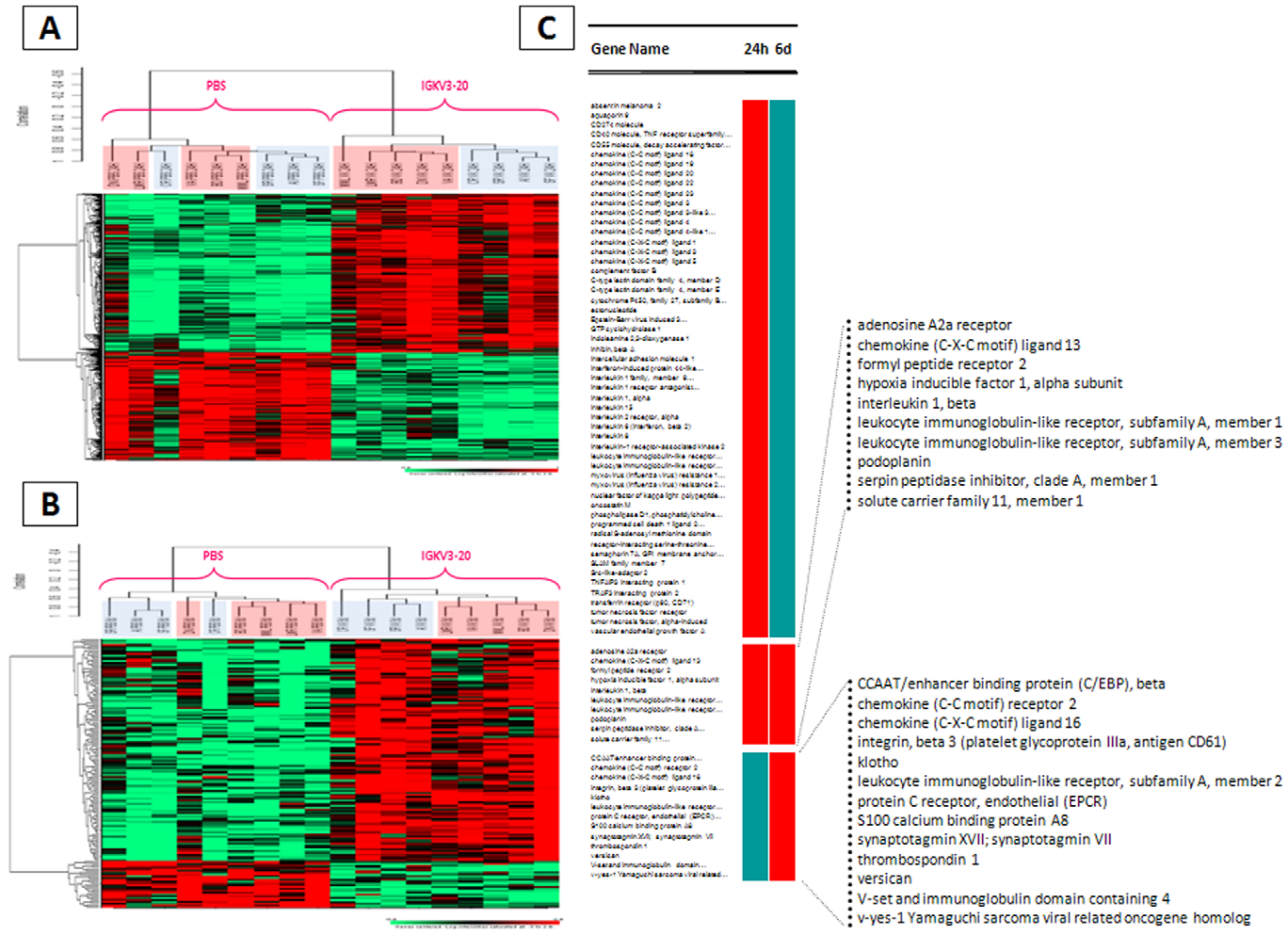
Supervised analysis based on IGKV3-20 induction. Heat map of gene sets differentially modulated in PBMCs by IGKV3-20 or PBS (unstimulated) after 24 h (**A**) or 6 d (**B**). HCV-positive subjects are highlighted in red. (**C**) Time course analysis on genes uniquely or commonly up-regulated at 24 h and 6 d time points. Up-regulated genes = red column.

The gene expression profile of PBMCs *ex vivo* stimulated with the IGKV3-20 protein was, subsequently, analyzed. Normalized microarray expression data were evaluated to assess the effect of treatment by supervised pair-wise comparisons between stimulated (IGKV3-20 treated) and non-stimulated (PBS) PBMCs, at 24 h (“early”) and 6 d (“late”), from both HCV-positive subjects and healthy controls (Fig. 4A and B).

The analysis identifies overall 503 genes differentially expressed at 24 h, of which 300 genes are up-regulated and 203 genes are down-regulated in IGKV3-20 stimulated PBMCs (Fig. 4A). Interestingly, unstimulated samples do not show a clear clustering according to the HCV positivity, while IGKV3-20 stimulated samples do (Fig. 4A). IGKV3-20 up-regulated genes were analyzed by Ingenuity Pathways Analysis, showing mostly inflammatory and immune related pathways at 24 h, while mostly atypical immune related pathways at 6 d (Fig. S1). Furthermore, genes up-regulated at 24 h were analyzed using the DAVID Bioinformatics Database (http://david.abcc.ncifcrf.gov) for associations with particular Gene Ontology (GO) terms. Among several functional annotations, those with the highest statistical significance are the Immune Response, the Inflammatory Response and the Defense Response. The 65 genes identified in the three annotations show a significant overlap, as indicated in the Table S1.

On the other hand, the same comparison analysis performed on PBMCs after 6 days incubation identifies 149 genes differentially expressed. Among them, 123 are up-regulated and 26 down-regulated in IGKV3-20 stimulated PBMCs which, also at this time point, show a clear clustering according to the HCV positivity (Fig. 4B). Up-regulated genes were further analyzed for association with GO terms, and among several annotations, those with the highest statistical significance are the Response to Wounding, the Inflammatory Response and the Defense Response. The 23 genes identified in such three annotations show a very significant overlap, as indicated in the Table S2. Interestingly, at 6 days incubation the Immune Response GO term is not represented at statistical significance.

The overall 88 genes up-regulated by IGKV3-20 from the considered functional annotations, have been used to perform a time-course analysis. The results show that while 55 (62.5%) genes are transiently activated at 24 h, 10 (11.3%) genes are steadily activated for 6 days and 13 (14.7%) genes are activated only at 6 days (Fig. 4C).

In particular, several leukocyte immunoglobulin-like receptors (LILRs) were steadily activated for 6 days (i.e. LILRA1 and LILRA3) or activated at 6 days (i.e. LILRA2), indicating a relevant role for such innate immune receptors in the response elicited by the IGKV3-20. In particular, overexpression of LILRA2 has been shown to induce a skewing towards a Th2-type cytokine profile, pushing the IL-10/IL-12 ratio in favor of IL-10 [36]. The time-course analysis provides, indeed, interesting clue on the *ex vivo* effect of IGKV3-20, suggesting a strong induction of an early innate immune response and a late pattern characterized by a prevalent B cell response.

In addition, the comparison between gene signatures induced by IGKV3-20 and LPS at 24 h and 6 d shows a significant overlapping of signatures induced by the two treatments at the two time points, which is instead very limited between “early” and “late” signatures induced by each of the treatments (Fig. S2).

### Genes activated by IGKV3-20 in PBMCs, based on HCV positivity status

In order to verify whether the underlying HCV infection may have an effect on the transcriptional profile of PBMCs induced by the IGKV3-20, a class-comparison analysis was performed selecting the two groups of subjects.

**Figure 5:**
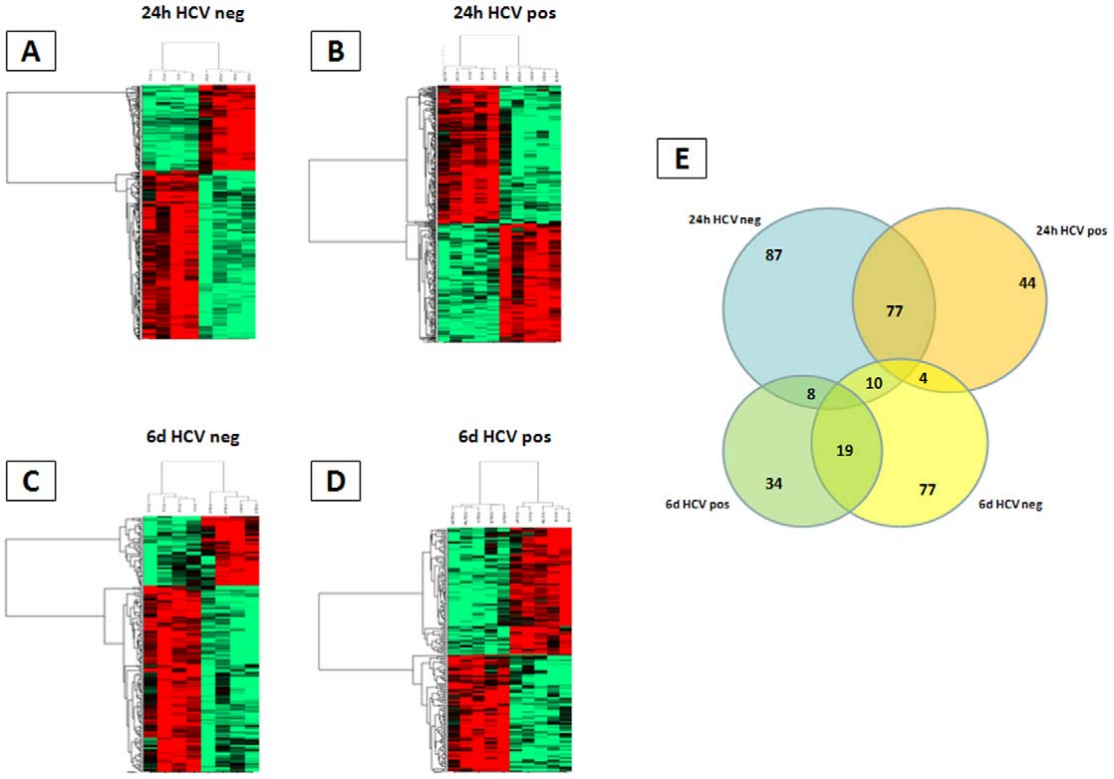
Supervised analysis based on IGKV3-20 induction in different sub-groups. (**A–D**) Heat map of gene sets differentially modulated in PBMCs by IGKV3-20 or PBS (unstimulated) in the four identified sub-groups. (**E**) Venn diagram indicating the number of unique and common up-regulated genes in the four sub-groups.

The analysis at 24 h on IGKV3-20 induced PBMCs in HCV-negative subjects identifies overall 329 genes differentially expressed, of which 218 are up-regulated and 111 are down-regulated; in HCV-positive subjects overall 310 genes are differentially expressed, of which 167 are up-regulated and 143 genes are down-regulated. Furthermore, the analysis at 6 d in HCV-negative subjects identifies overall 180 genes differentially expressed, of which 129 are up-regulated and 51 are down-regulated; in HCV-positive subjects overall 183 genes are differentially expressed, of which 96 are up-regulated and 87 genes are down-regulated (Fig. 5 A–D).

The genes up-regulated by IGKV3-20 treatment at 24 h and 6 d in PBMCs from both HCV-negative and positive subjects have been compared to identify shared and unique signature patterns. Several genes are common to the specific subsets, whereas a significant number of genes are unique and associated with several functional annotations, including cytokine, inflammatory and defense response (Fig. 5 E and Tables S3, S4, S5, S6).

Notably, the innate inhibitory receptors LILRB1, LILRB2 are among the unique genes up-regulated by IGKV3-20 in PBMCs from HCV-positive subjects at 6 d. Both receptors have been shown to inhibit APC response to maturation stimuli, including an altered cytokine secretion profile, which results in hampered CD4^+^ T helper cell response [37], [38], [39], [40].

Taken together, these findings would suggest that the response to IGKV3-20 in HCV-positive subjects is characterized by a possible impairment of the CD4^+^ T helper cells.

### Ingenuity Pathways Analysis on IGKV3-20 induced genes

The Ingenuity Pathways Analysis (IPA) was performed to understand the biological role of genes differentially expressed in the supervised comparisons and human pathway lists were determined by “Ingenuity System Database”, setting the significance threshold of t-test at p<0.05.

The pathways activated at 24 h in both groups of samples are significantly overlapping and are mostly related to inflammation and innate immunity; however, it is noteworthy that in HCV-positive samples the top ranking pathways include those related to LXR/RXR Activation and Oncostatin M Signaling, which are not present in HCV-negative samples (Fig. S3 A and B). The comparison of “early” (24 h) and “late” (6 d) pathways, instead, shows very narrow overlapping, limited to the inflammatory-related pathways, mainly considering the HCV-negative samples. The “late” pathways in both groups of samples show very limited overlapping, with prevalent activation of “atypical” immune related pathways. Of note, the HCV-negative samples show the activation of TLR Signaling and B Cell Activating Factor Signaling (Fig. S3 C and D).

Among the pathways identified by IPA analysis, based on up-regulated genes in PBMCs treated with IGKV3-20 for 24 h and 6 days, gene networks relevant to the present study, have been subsequently selected. In particular, the network of Communication between Innate and Adaptive Immunity confirms a higher number of up-regulated genes at 24 h compared to 6 days. Moreover, limited number of genes are differentially modulated between samples from HCV-negative and positive subjects. These latter include the NF-kB complex, which is up-regulated only at 24 h in HCV-negative samples; the LXR (NR1H3), up-regulated only at 24 h in HCV-positive subjects; JUN and FOS, up-regulated only at 6 d in HCV-negative samples; and IL-17A, up-regulated only at 6 d in HCV-positive samples (Fig. S4, S5, S6, S7).

### Integrated analysis of the immune signatures induced in PBMCs by recombinant IGKV3-20 protein

The large number of immunological genes and pathways modulated by IGKV3-20 has prompted us to investigate in more detail their specific role.

**Figure 6:**
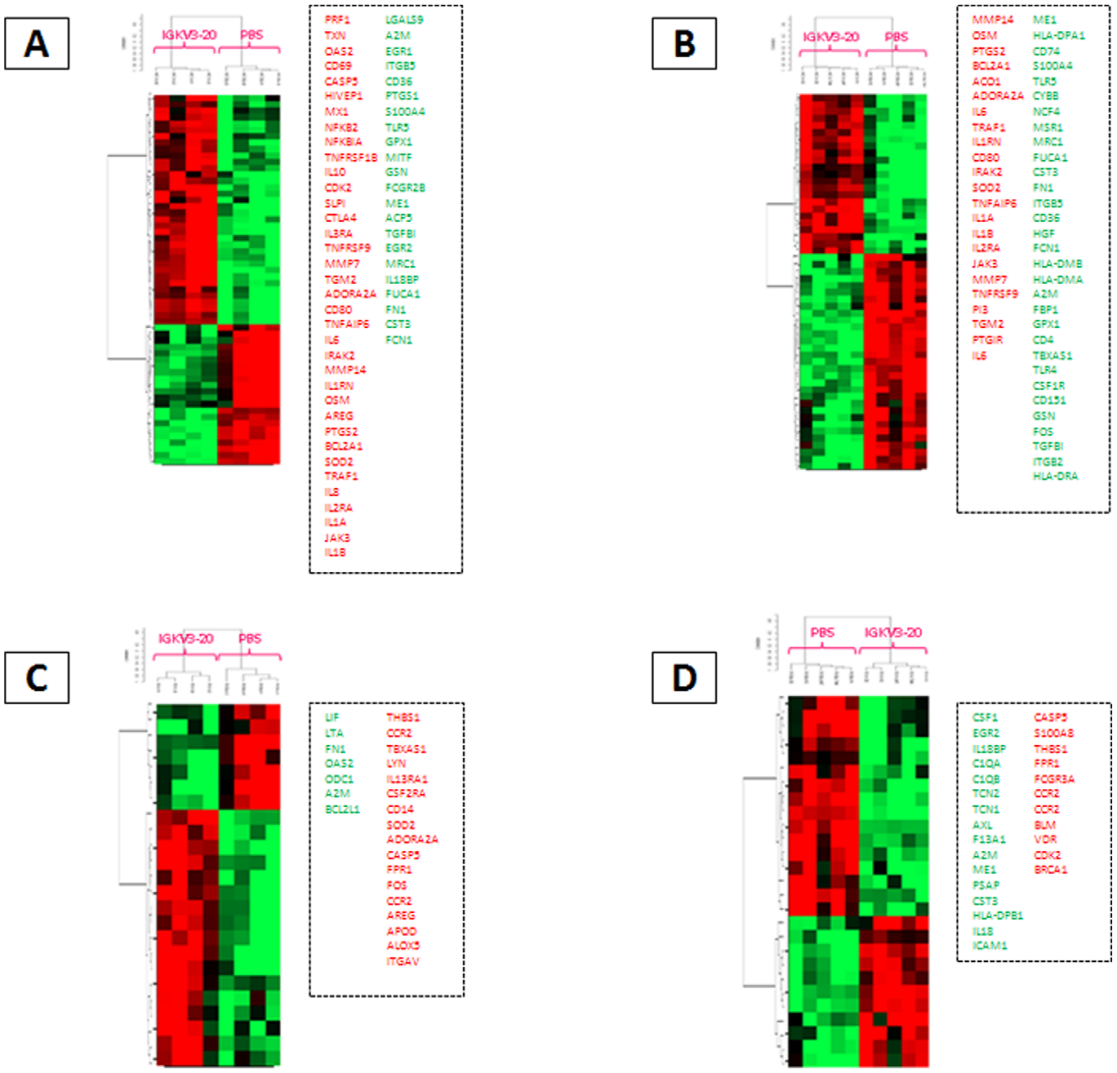
Supervised analysis based on IGKV3-20 induction of immunology genes in different sub-groups. Heat map of immunology gene sets differentially modulated in PBMCs by IGKV3-20 or PBS (unstimulated). Genes up-regulated (red) and down-regulated (green) by IGKV3-20 are listed for each analysis. (**A**) 24 h HCV-negative; (**B**) 24 h HCV-positive; (**C**) 6 d HCV-negative; (**D**) 6 d HCV-positive.

To this aim, normalized microarray expression data were evaluated by pair-wise comparison between IGKV3-20-stimulated and PBS-stimulated PBMCs, using specific CGAP (Cancer Genome Anatomy Project) immunology gene lists for supervised analysis. The evaluation was performed comparing the immune gene signatures modulated at 24 h and 6 d in HCV-negative and positive samples (Fig. 6A–D). The analysis shows that 24 h induction with IGKV3-20 induces a modulation of 58 immune genes in HCV-negative samples (36 up and 22 down regulated) and 54 in HCV-positive samples (23 up and 31 down regulated) (Fig. 6A and B). On the other hand, after 6 days, IGKV3-20 modulates the induction of 24 genes in HCV-negative samples (17 up and 7 down regulated) and of 27 in HCV-positive samples (11 up and 16 down regulated) (Fig. 6C and D).

**Figure 7:**
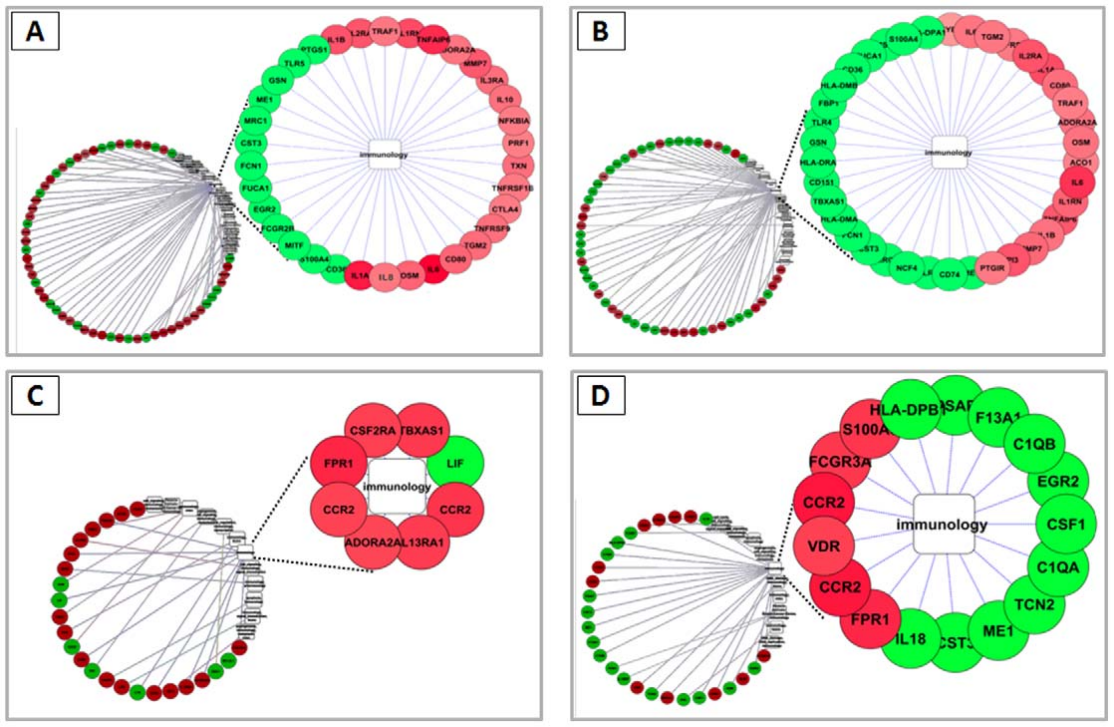
Cytoscape analysis. Integrated analysis of immunology genes differentially modulated in PBMCs by IGKV3-20. Genes up-regulated are indicated in red and down-regulated are indicated in green. (**A**) 24 h HCV-negative; (**B**) 24 h HCV-positive; (**C**) 6 d HCV-negative; (**D**) 6 d HCV-positive.

The resulting immune networks were visualized using Cytoscape (http://www.cytoscape.org), an open source software for complex network analysis and visualization (Fig. 7 A–D).

Among the different identified networks, the majority of genes group together in the “immunology” network, which includes a higher number of modulated genes for PBMCs incubated with IGKV3-20 for 24 h (Fig. 7 A and B) than for PBMCs incubated for 6 days (Fig. 7 C and D). The whole set of identified immune networks is represented in the supplementary materials (Fig. S8, S9, S10, S11).

**Figure 8:**
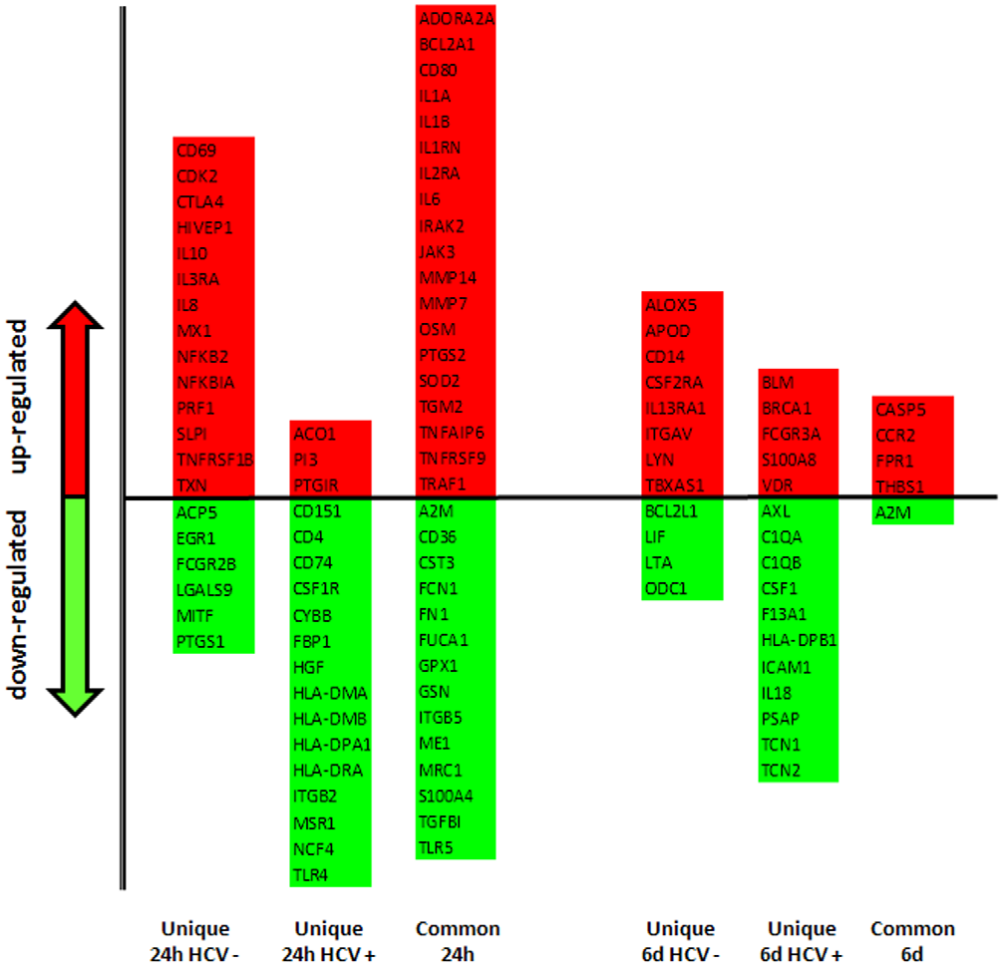
Analysis of immunology genes induced by IGKV3-20. Immunology genes differentially modulated in PBMCs by IGKV3-20, listed as unique and common in the indicated sub-groups.

Among the genes up and down regulated at the two time-points, unique and common immune signatures are identified in the HCV-negative and positive samples (Fig. 8). In particular, at 24 h post-induction, it is observed a significant up-regulation of genes of cytokine and cytokine receptor families, as well as of downstream signaling cascades, including IL-1, IL-6, OSM (LIF), IL-10, TNFRSF1B [41], TNFAIP6 [42], IRAK2 [43], TRAF1 [44] and JAK3 [45].

Other mediators of innate immunity (i.e. IL-8) and pro-inflammatory cytokines (i.e. IL-6) are up-regulated at 24 h by IGKV3-20 in both group of samples (Fig. 8). Moreover, gene signatures suggesting the activation of an early adaptive immune response are found up-regulated, including the IL-2 receptor alpha (IL2RA) and the mediators of T cell survival cyclin-dependent kinase 2 (CDK2) and BCL2 (BCL2A1) [46].

The evidence of the up-regulation of several surface activation markers of the immune cells, such as IL3RA (CD123), CD80, CD69 and tumor necrosis factor receptor superfamily member 9 (TNFRSF9) [47,47], confirms our preliminary results on the ability of IGKV3-20 to induce the expression of activation markers and co-stimulatory molecules on circulating APCs after 24 h incubation [26].

On the other hand, at the same time point, it is observed an anti-inflammatory gene signature characterized by up-regulation of IL-10, Interleukin 1 receptor antagonist (IL1RN), and down-regulation of genes crucial for the antigen processing and presentation pathway via MHC class-II (i.e. CD4, CD74, HLA-DMA, HLA-DMB, HLA-DPA1, HLA-DRA), the latter genes only in the HCV-positive samples (Fig. 8).

At 6 d post-induction, the overall number of modulated genes is drastically reduced. Among the up-regulated genes, their function are mainly attributable to defense response, including pathogen recognition and immune response (FPR1, CD14, LYN, FCGR3A), chemokine and cytokine receptors (CCR2 and IL13RA1), growth factors (CSFR2A), arachidonate metabolism (ALOX5 and VDR), S100 calgranulins (S100A8) (Fig. 8).

On the other hand, among the few down-regulated genes the most relevant to the present study are IL18 and HLA-DPB1, observed in the HCV-positive samples. This would suggest, for such sub-group, a possible impairment of the CD4^+^ T helper cells function, and in particular of the differentiation into Th1 cells, also at the “late” time point [48] (Fig. 8).

## Discussion

In the present study, we report the results of a multivariate and multiparametric analysis of the innate and early adaptive immune response induced in PBMCs, from HCV-positive and negative subjects, by a candidate idiotype vaccine for HCV-related B cell disorders. To this aim, PBMCs were treated *ex vivo* with the IGKV3-20 light-chain protein idiotype and the immune signature was assessed by analysis of cell surface activation molecules, cytokine profile as well as whole gene transcriptome.

The analysis of surface activation markers has shown that basal and IGKV3-20-induced expression is comparable in the two group of subjects in both CD14^+^ and CD11c^+^ cell populations. The overall expression pattern suggested a maturation/activation induced by IGKV3-20 and a significant induction of CD80 expression was observed in CD11c^+^ cell population from the HCV-negative subjects (Fig. 1). Such pattern was also induced by LPS, used as a positive activation factor. This observation suggests that the HCV positivity impairs neither the immune activation status nor the responsiveness of circulating APC populations to an immunogenic stimulus, and confirms data from others showing a normal expression of surface molecules on immature and mature DCs from HIV-1-infected and HCV/HIV-co-infected individuals [49], [50], [51].

The cytokine pattern for IL-2, IL-5, IL-6, IL-10, IFN-γ and TNF-α was assessed by ELISA in supernatant of stimulated PBMCs, at 24 h or 6 days of incubation, showing a significant persistent induction of IL-6 and a transient induction for IL-10 and TNF-α. No activation of Th1 cytokines (IL-2 and IFN-γ) or of additional Th2 cytokines (IL-5) was observed, in agreement with previous results also by our group [26], [52]. Such results indicate that IGKV3-20 induces a prevalent Th2-biased cytokine pattern, similar to LPS but less potent, with no significant differences between HCV-negative and positive subjects (Fig. 2). The induction of an anti-inflammatory molecule, such as IL-10, suggests the activation of a positive homeostatic mechanism to counteract an excessive pro-inflammatory condition which would potentially be detrimental to an effective immune response.

The global gene expression profile of samples from healthy controls and HCV-positive subjects exposed to IGKV3-20 was analyzed. The preliminary unsupervised analysis showed a clear cut clustering of samples according to HCV positivity status and duration (24 h vs 6 d) of treatment (Fig. 3). A subsequent supervised analysis was performed by pair-wise comparison between stimulated and unstimulated PBMCs to identify gene signatures and pathways induced by the IGKV3-20. The most relevant changes occurred at 24 h and resolved by 6 d; the analysis, indeed, identified 503 genes differentially expressed after 24 h and 149 genes after 6 d incubation, with a statistically significant segregation of samples according to their HCV positivity status (Fig. 4). The IPA analysis performed on “early” and “late” up-regulated genes showed a prevalence of inflammation and innate immunity-related pathways activated at 24 h, with significant overlapping between HCV groups, and a prevalence of “atypical” immune pathways activated at 6 d, with poor overlapping between HCV groups. Such results suggest that, irrespective to the HCV status, IGKV3-20 at 24 h is able to induce a productive early immune activation, paving the way to an effective adaptive immune response, which is further supported by the finding at 6 d of activated pathways related to TLR Signaling and B Cell Activating Factor Signaling (Fig. S3).

Interestingly, the LXR/RXR pathway was found up-regulated uniquely and in top ranking position in HCV-positive samples at 24 h, in agreement with data showing that such pathway is involved in the increased lipogenesis and, consequently, steatosis which characterizes progression of HCV chronic infection [53], [54].

A detailed analysis was performed on “early” and “late” gene signatures associated to immune pathways induced by IGKV3-20 in PBMCs from both HCV-negative and positive subjects. To this aim, a specific CGAP immunology gene list was used for supervised analysis and a limited set of modulated genes were identified in each defined sample sub-group (Fig. 6). Up-regulated genes were assigned to individual immune networks using Cytoscape for visualization, and gene functions were identified according to DAVID Bioinformatics Database (Fig. 7 and Fig. S8, S9, S10, S11). A significantly higher number of modulated genes was identified in PBMCs incubated with IGKV3-20 for 24 h (Fig. 7 A and B) than in those incubated for 6 days and unique as well as common immune signatures were identified in the HCV-negative and positive samples at the two time-points (Fig. 8).

Indeed, at 24 h post-induction, a significant up-regulation of genes of cytokine and cytokine receptor families, as well as of downstream signaling cascades, was observed. In particular, both IL1A and IL1B are up-regulated after 24 h in both HCV-negative and positive samples. IL-1 cytokine is known to be produced by activated macrophages, it is an important mediator of the inflammatory response and is involved in several cellular activities, including cell proliferation, differentiation and apoptosis [55]. IL-1 shares with TNF-α similar downstream pathways, involving the so called TNF receptor associated factors (TRAFs), which ultimately lead to activation of NF-kB transcriptional factor for triggering their pro-inflammatory activity [56].

Our results, indeed, strongly suggest an IGKV3-20-mediated activation of IL-1 and TNF-α signaling, as indicated by the up-regulation of several genes involved in such specific pathways, including the interleukin-1 receptor-associated kinase-like 2 (IRAK2), which is associated with the IL-1 receptor (IL1R) upon stimulation [43] and TNF receptor-associated factor 1 (TRAF1), which forms a heterodimeric complex with TRAF2, which is required for TNF-alpha-mediated activation of MAPK8/JNK and NF-kB [57], [58], [44].

Furthermore, several genes specifically induced by IL-1 and TNF-α are up-regulated by IGKV3-20 stimulation, including tumor necrosis factor alpha-induced protein 6 (TNFAIP6), which plays a relevant role in protease network associated with inflammation [42] and BCL2-related protein A1 (BCL2A1), which is a direct transcription target of NF-kB [59]. Finally, NFKB2 subunit was found to be up-regulated, as well. Furthermore, the up-regulation of IL1B and associated genes (i.e. CASP5, IL1RN) strongly suggests the activation of the inflammasome by IGKV3-20 at 24 h in both HCV groups, which represents one of the main pathways of the innate immune response [60], [61], [31], [62].

Signatures suggesting the activation of an early adaptive immune response are found up-regulated as well, including the IL-2 receptor alpha (IL2RA), which is known to mediate the IL-2 biological effect on T cell growth, differentiation and survival via induction of a set of genes, such as cyclin E, which binds G1-phase cyclin-dependent kinase 2 (CDK2), and BCL2 (BCL2A1), which is required for T cell survival [46]. Indeed, both CDK2 and BCL2A1 are found up-regulated in our analysis. The up-regulation of JAK3 further supports the activation of the IL-2/IL2RA axis, considering its central role as mediator of cytokine signaling transduction via type I cytokine receptor family (i.e. IL-2R, IL-4R, IL-7R, IL-9R, IL-15R) [63] in T cells and NK cells, in which JAK3 is mostly expressed.

Moreover, several surface activation markers of the immune cells, such as IL3RA (CD123), CD80, CD69 and tumor necrosis factor receptor superfamily member 9 (TNFRSF9) [47,47], are activated by IGKV3-20. Indeed, CD80 molecule, found on activated B cells, as well as monocytes, is able to provide a costimulatory signal necessary for T cell activation and survival [64]. Moreover, the activation of T cells, both in vivo and in vitro, is known to induce the expression of CD69, which appears to be the earliest inducible cell surface glycoprotein acquired during lymphoid activation [65], being involved in T cell proliferation [66].

Finally, TNFRSF9 (CD137) is known to be expressed by activated T cells, DCs, follicular dendritic cells, natural killer cells, granulocytes and cells of blood vessel walls at sites of inflammation [67]. TNFRSF9 is known to play a key role in the survival of activated and memory CD8^+^ T cells, upon interaction with TNFR associated factors (TRAFs), including TRAF1, enhancing T cell proliferation, IL-2 secretion and cytolytic activity (reviewed in [68]).

Most of the up-regulated genes are shared between the two HCV groups of samples, suggesting that, at least in our cohort of subjects, the HCV infection does not impair the response of the immune system to the IGKV3-20 candidate idiotypic vaccine. HCV-negative subjects show unique up-regulated gene signature indicating a pronounced inflammatory status and a counterbalanced T cell activation. Indeed, both PRF1 (perforin 1), which is a key player in the secretory granule-dependent cell death [69], and CTLA4 (cytotoxic T-lymphocyte-associated protein 4), which is a major negative regulator of T-cell responses [70], are up-regulated.

Overall, such “early” gene signatures identified by gene expression profiling analysis are in striking agreement with both marker expression (CD80) data obtained by flow cytometry analysis and cytokine pattern (IL-6, IL-10, TNF-related pathway), confirming the sensibility, specificity and biological significance of the gene transcriptional profiling.

At 6 d post-induction, the overall number of modulated genes is drastically reduced but still indicative of an adaptive B cell response, in both HCV-negative and positive sub-groups. In particular, among the up-regulated genes, the tyrosine-protein kinase (LYN) has been recognized as a key player in the signal transduction for expansion and differentiation of peripheral B cells [71] and the Fc fragment of IgG, low affinity IIIa, receptor - CD16a (FCGR3A) is known to mediate antibody-dependent cellular cytotoxicity (ADCC), as well as other antibody-dependent responses, such as phagocytosis [72], [73]. The interleukin 13 receptor - alpha 1 (IL13RA1) is a subunit of the interleukin 13 receptor, which forms a complex with IL-4 receptor alpha and is involved in mediating the signaling processes that lead to the activation of JAK1, STAT3 and STAT6 induced by either IL13 or IL4 [74], [75]. Such mediator activity is accomplished upon binding to tyrosine kinase TYK2, which has been shown to interact also with LYN [76]. The chemokine (C-C motif) receptor 2 (CCR2) is a receptor for monocyte chemoattractant protein-1, which mediates monocyte chemotaxis and infiltration in inflammatory sites [77].

Moreover, several members of the Leukocyte immunoglobulin (Ig)-like receptor (LILR) family have been found up-regulated mainly in HCV-positive subjects. LILRs are part of the innate immune receptors and their genes are expressed by immune cell types of both myeloid and lymphoid lineages. They exert powerful immunomodulatory effects on innate and adoptive immune responses [78], [79]. Our results confirm that LILRs may play a key role in the immune response to vaccines, mainly regulating antibody responses, as recently reported [80].

Overall, the multiparametric analysis performed on PBMCs loaded *ex vivo* with the IGKV3-20 candidate idiotypic vaccine shows, irrespective of the HCV status, an early (24 h) gene expression pattern characterized by the strong induction of an inflammatory and innate immune response and a late (6 d) pattern characterized by a prevalent B cell response. On the contrary, induction of Th1 cellular immune response appears to be negligible. Moreover, several up-regulated genes suggest a possible inhibitory effect on CD4^+^ T helper cells, which may result in an inefficient establishment of immune memory to the vaccine.

Such results may have a potential implication on the implementation of the idiotype vaccine based on the IGKV3-20 protein, suggesting that 1) HCV positivity does not impair the efficacy of immune response; 2) a specific Th1-driving adjuvant formulation should be adopted to elicit therapeutically relevant immune responses; 3) innate immune inhibitory signals should be counteracted to improve CD4^+^ T helper cell activation for amplifying effector immune response and establishing a memory cell population.

In conclusion, our results strongly suggest the usefulness of multiparametric analysis to identify predictive markers of the immune response to a candidate vaccine molecule, which would represent an invaluable tool for vaccine development and optimization. Additional studies on larger cohorts and comparison with data obtained on cells from *in vivo* vaccinated subjects will further validate such predictive markers.

## Supporting Information

Figure S1 **Ingenuity Pathways Analysis.** Analysis of canonical pathways differentially up-regulated in PBMCs by IGKV3-20 at 24 h (**A**) and 6 d (**B**) without distinction between HCV subgroups. Statistical significance is expressed as negative logarithm (-log).
(TIF)

Figure S2 **Analysis of immunology genes induced by IGKV3-20 and LPS.** Venn diagram indicating the number of unique and common up-regulated genes in the identified four sub-groups, without distinction according to HCV positivity.
(TIF)

Figure S3 **Ingenuity Pathways Analysis.** Analysis of canonical pathways differentially up-regulated in PBMCs by IGKV3-20 at the two time points in HCV subgroups. (**A**) 24 h HCV-negative; (**B**) 24 h HCV-positive; (**C**) 6 d HCV-negative; (**D**) 6 d HCV-positive. Statistical significance is expressed as negative logarithm (-log).
(TIF)

Figure S4 **Dynamic network of genes differentially induced by IGKV3-20 at 24 h in HCV-negative samples.** Network of genes involved in the communication between innate and adaptive immune cells. Genes up-regulated in PBMCs by IGKV3-20 are shown in red. The networks were generated through the use of Ingenuity Pathways Analysis (Ingenuity Systems, www.ingenuity.com).
(TIF)

Figure S5 **Dynamic network of genes differentially induced by IGKV3-20. at 24 h in HCV-positive samples.** Network of genes involved in the communication between innate and adaptive immune cells. Genes up-regulated in PBMCs by IGKV3-20 are shown in red. The networks were generated through the use of Ingenuity Pathways Analysis (Ingenuity Systems, www.ingenuity.com).
(TIF)

Figure S6 **Dynamic network of genes differentially induced by IGKV3-20 at 6 d in HCV-negative samples.** Network of genes involved in the communication between innate and adaptive immune cells. Genes up-regulated in PBMCs by IGKV3-20 are shown in red. The networks were generated through the use of Ingenuity Pathways Analysis (Ingenuity Systems, www.ingenuity.com).
(TIF)

Figure S7 **Dynamic network of genes differentially induced by IGKV3-20 at 6 d in HCV-positive samples.** Network of genes involved in the communication between innate and adaptive immune cells. Genes up-regulated in PBMCs by IGKV3-20 are shown in red. The networks were generated through the use of Ingenuity Pathways Analysis (Ingenuity Systems, www.ingenuity.com).
(TIF)

Figure S8 **Cytoscape analysis of genes modulated by IGKV3-20 at 24 h in HCV-negative samples.** Integrated analysis of immune genes differentially modulated in PBMCs by IGKV3-20. Genes up-regulated are indicated in red and down-regulated are indicated in green.
(TIF)

Figure S9 **Cytoscape analysis of genes modulated by IGKV3-20 at 24 h in HCV-positive samples.** Integrated analysis of immune genes differentially modulated in PBMCs by IGKV3-20. Genes up-regulated are indicated in red and down-regulated are indicated in green.
(TIF)

Figure S10 **Cytoscape analysis of genes modulated by IGKV3-20 at 6 d in HCV-negative samples.** Integrated analysis of immune genes differentially modulated in PBMCs by IGKV3-20. Genes up-regulated are indicated in red and down-regulated are indicated in green.
(TIF)

Figure S11 **Cytoscape analysis of genes modulated by IGKV3-20 at 6 d in HCV-positive samples.** Integrated analysis of immune genes differentially modulated in PBMCs by IGKV3-20. Genes up-regulated are indicated in red and down-regulated are indicated in green.
(TIF)

Table S1 **List of genes up-regulated in PBMCs by IGKV3-20 at 24 h.** The genes of Gene Ontology terms with the highest statistical significance (<10^−16^) are listed and their presence in each term is annotated.
(DOC)

Table S2 **List of genes up-regulated in PBMCs by IGKV3-20 at 6 d.** The genes of Gene Ontology terms with the highest statistical significance (<10-8) are listed and their presence in each term is annotated.
(DOC)

Table S3 **List of unique genes up-regulated by IGKV3-20 in PBMCs from HCV negative subjects at 24 h.**
(DOC)

Table S4 **List of unique genes up-regulated by IGKV3-20 in PBMCs from HCV positive subjects at 24 h.**
(DOC)

Table S5 **List of unique genes up-regulated by IGKV3-20 in PBMCs from HCV negative subjects at 6 d.**
(DOC)

Table S6 **List of unique genes up-regulated by IGKV3-20 in PBMCs from HCV positive subjects at 6 d.**
(DOC)
