# Supplementary figures and images for: Multiparametric Analyses of Human PBMCs Loaded Ex Vivo with a Candidate Idiotype Vaccine for HCV-Related Lymphoproliferative Disorders
Source: PLoS One. 2012 Sep 18;7(9):e44870. doi: 10.1371/journal.pone.0044870 (PMC3445594; doi:10.1371/journal.pone.0044870)

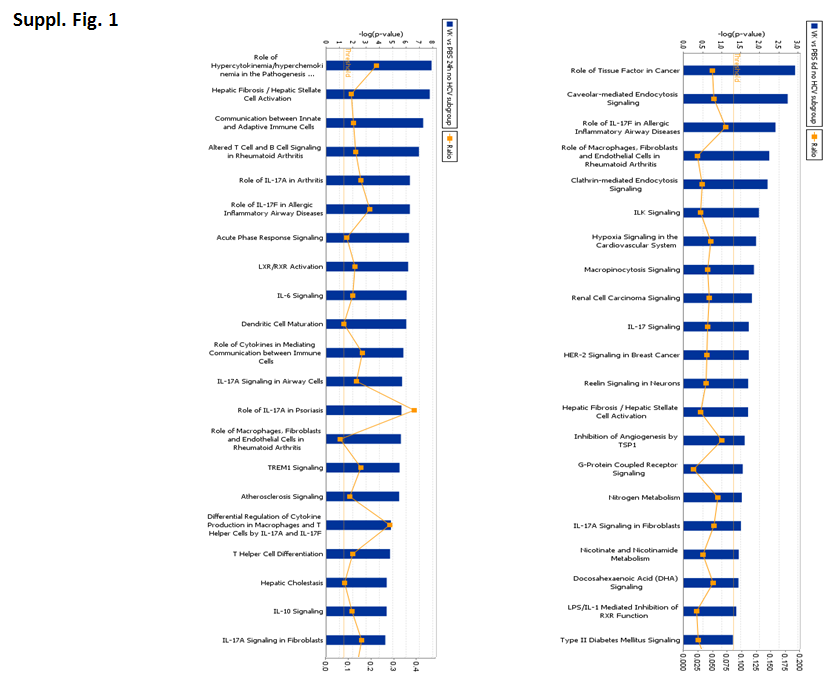

Supplement: Figure S1 — Ingenuity Pathways Analysis. Analysis of canonical pathways differentially up-regulated in PBMCs by IGKV3-20 at 24 h (A) and 6 d (B) without distinction between HCV subgroups. Statistical significance is expressed as negative logarithm (-log). (TIF) [file pone.0044870.s001.tif]

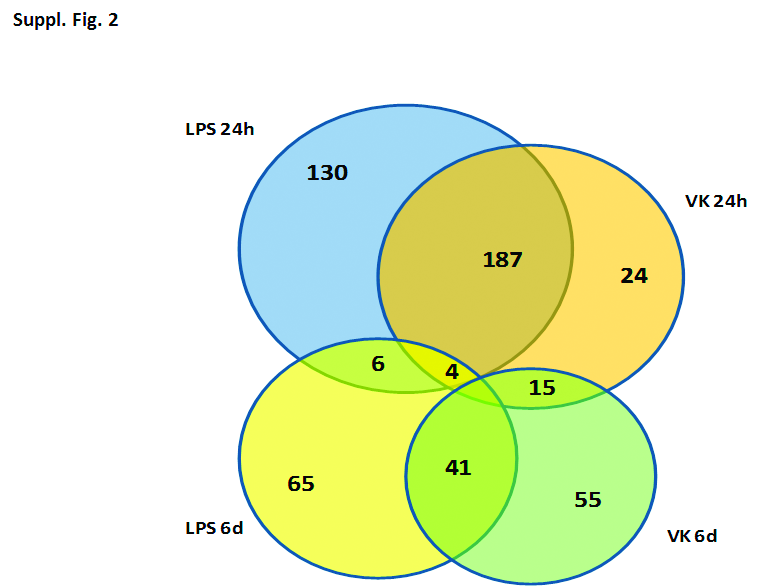

Supplement: Figure S2 — Analysis of immunology genes induced by IGKV3-20 and LPS. Venn diagram indicating the number of unique and common up-regulated genes in the identified four sub-groups, without distinction according to HCV positivity. (TIF) [file pone.0044870.s002.tif]

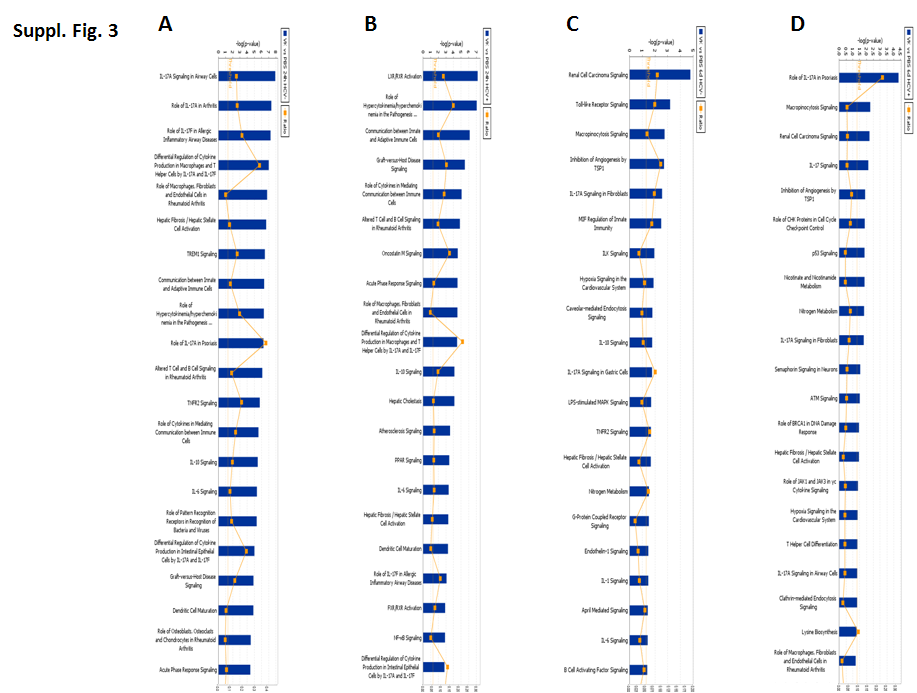

Supplement: Figure S3 — Ingenuity Pathways Analysis. Analysis of canonical pathways differentially up-regulated in PBMCs by IGKV3-20 at the two time points in HCV subgroups. (A) 24 h HCV-negative; (B) 24 h HCV-positive; (C) 6 d HCV-negative; (D) 6 d HCV-positive. Statistical significance is expressed as negative logarithm (-log). (TIF) [file pone.0044870.s003.tif]

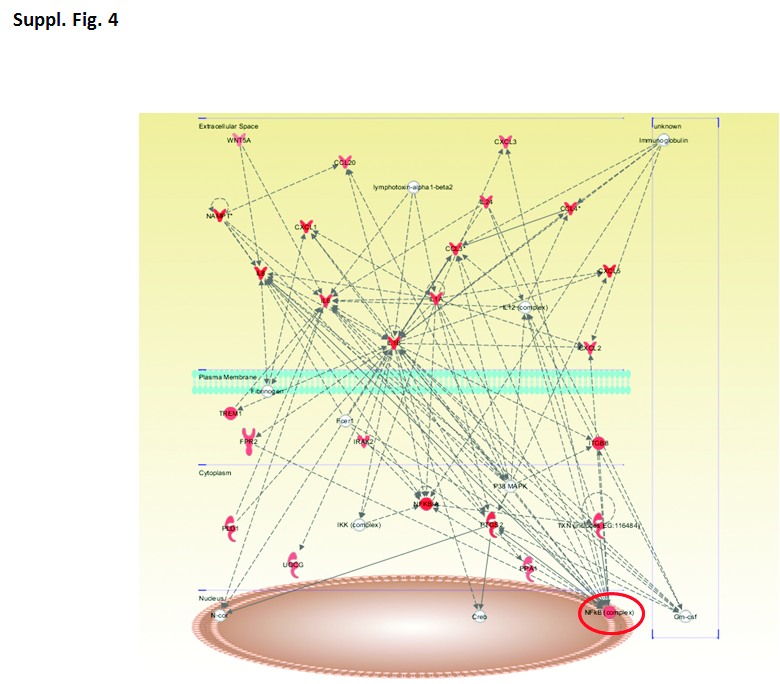

Supplement: Figure S4 — Dynamic network of genes differentially induced by IGKV3-20 at 24 h in HCV-negative samples. Network of genes involved in the communication between innate and adaptive immune cells. Genes up-regulated in PBMCs by IGKV3-20 are shown in red. The networks were generated through the use of Ingenuity Pathways Analysis (Ingenuity Systems, www.ingenuity.com). (TIF) [file pone.0044870.s004.tif]

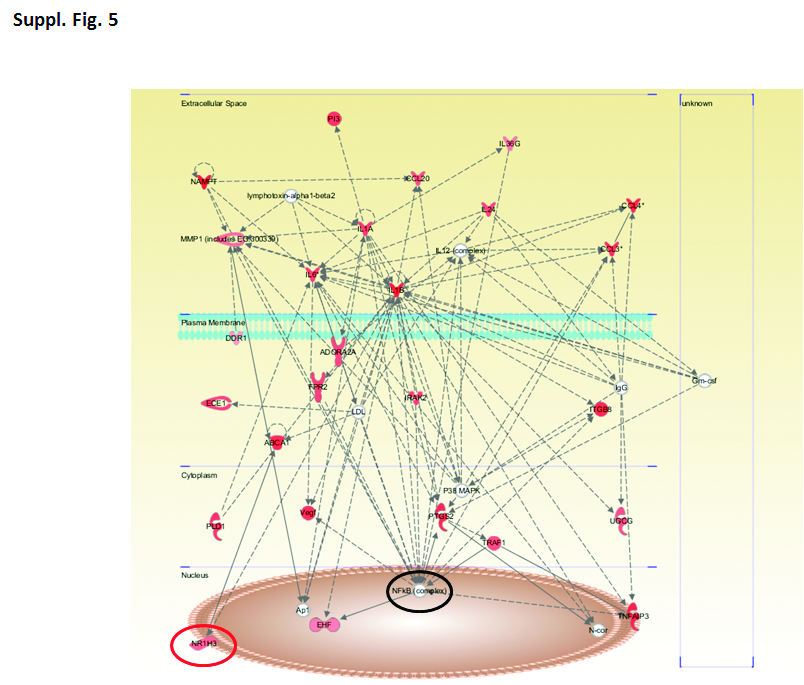

Supplement: Figure S5 — Dynamic network of genes differentially induced by IGKV3-20. at 24 h in HCV-positive samples. Network of genes involved in the communication between innate and adaptive immune cells. Genes up-regulated in PBMCs by IGKV3-20 are shown in red. The networks were generated through the use of Ingenuity Pathways Analysis (Ingenuity Systems, www.ingenuity.com). (TIF) [file pone.0044870.s005.tif]

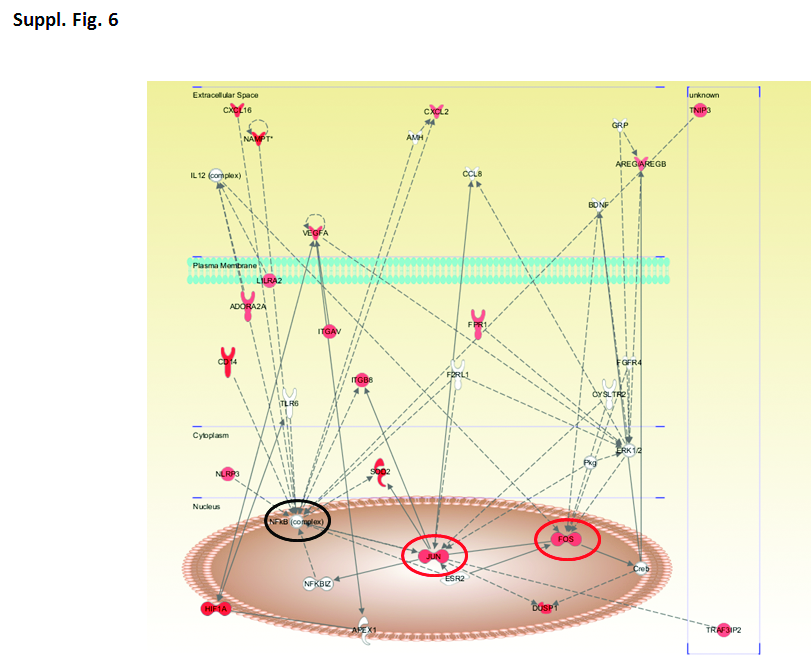

Supplement: Figure S6 — Dynamic network of genes differentially induced by IGKV3-20 at 6 d in HCV-negative samples. Network of genes involved in the communication between innate and adaptive immune cells. Genes up-regulated in PBMCs by IGKV3-20 are shown in red. The networks were generated through the use of Ingenuity Pathways Analysis (Ingenuity Systems, www.ingenuity.com). (TIF) [file pone.0044870.s006.tif]

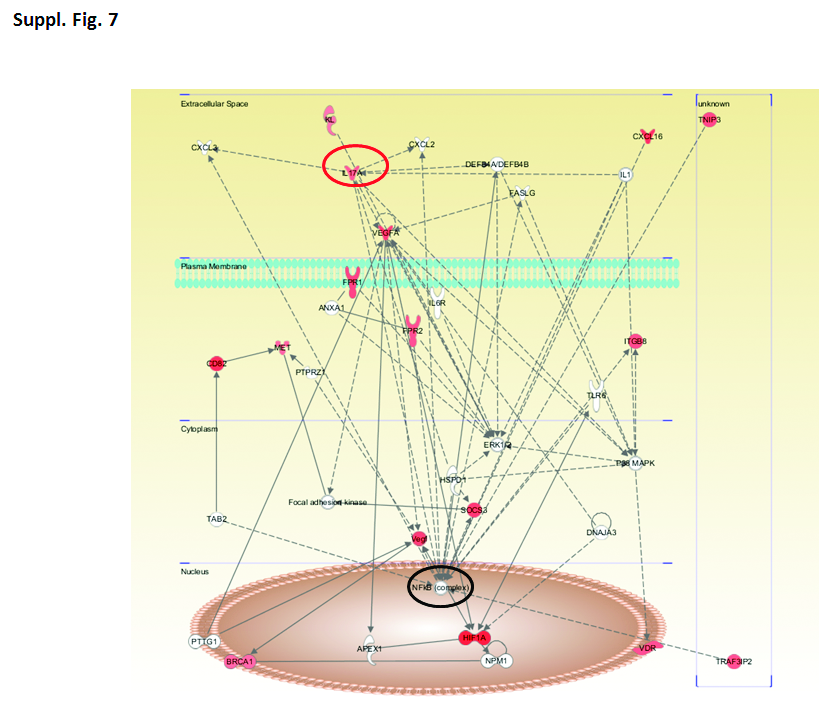

Supplement: Figure S7 — Dynamic network of genes differentially induced by IGKV3-20 at 6 d in HCV-positive samples. Network of genes involved in the communication between innate and adaptive immune cells. Genes up-regulated in PBMCs by IGKV3-20 are shown in red. The networks were generated through the use of Ingenuity Pathways Analysis (Ingenuity Systems, www.ingenuity.com). (TIF) [file pone.0044870.s007.tif]

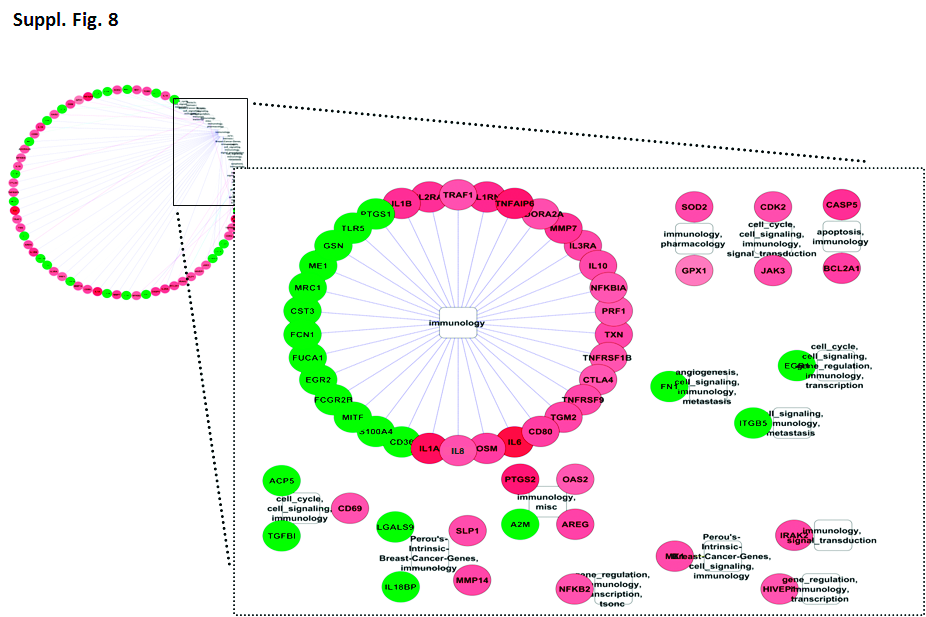

Supplement: Figure S8 — Cytoscape analysis of genes modulated by IGKV3-20 at 24 h in HCV-negative samples. Integrated analysis of immune genes differentially modulated in PBMCs by IGKV3-20. Genes up-regulated are indicated in red and down-regulated are indicated in green. (TIF) [file pone.0044870.s008.tif]

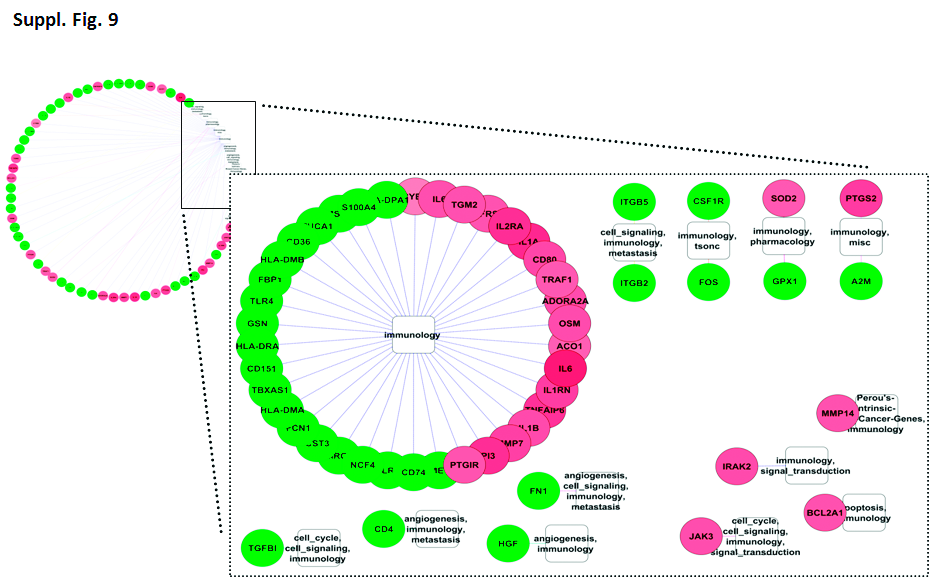

Supplement: Figure S9 — Cytoscape analysis of genes modulated by IGKV3-20 at 24 h in HCV-positive samples. Integrated analysis of immune genes differentially modulated in PBMCs by IGKV3-20. Genes up-regulated are indicated in red and down-regulated are indicated in green. (TIF) [file pone.0044870.s009.tif]

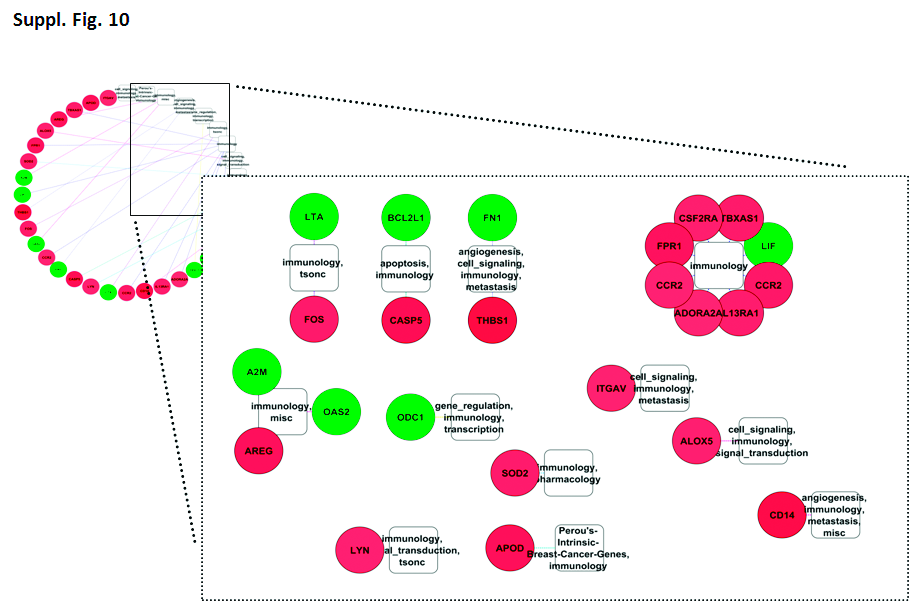

Supplement: Figure S10 — Cytoscape analysis of genes modulated by IGKV3-20 at 6 d in HCV-negative samples. Integrated analysis of immune genes differentially modulated in PBMCs by IGKV3-20. Genes up-regulated are indicated in red and down-regulated are indicated in green. (TIF) [file pone.0044870.s010.tif]

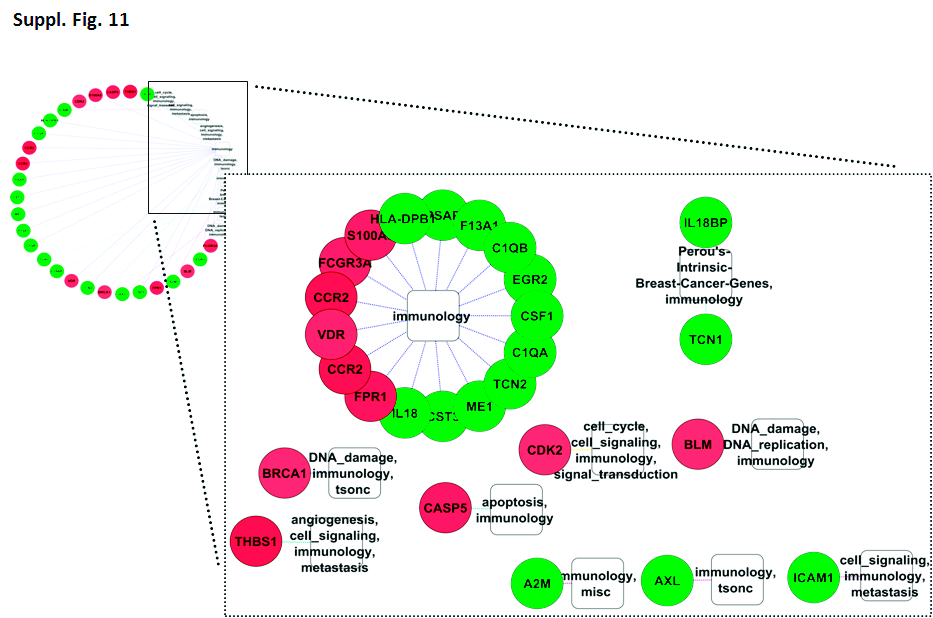

Supplement: Figure S11 — Cytoscape analysis of genes modulated by IGKV3-20 at 6 d in HCV-positive samples. Integrated analysis of immune genes differentially modulated in PBMCs by IGKV3-20. Genes up-regulated are indicated in red and down-regulated are indicated in green. (TIF) [file pone.0044870.s011.tif]
