# Supplementary material for: Multiparametric Analyses of Human PBMCs Loaded Ex Vivo with a Candidate Idiotype Vaccine for HCV-Related Lymphoproliferative Disorders
Source: PLoS One. 2012 Sep 18;7(9):e44870. doi: 10.1371/journal.pone.0044870 (PMC3445594; doi:10.1371/journal.pone.0044870)
Supplement: Table S1 — List of genes up-regulated in PBMCs by IGKV3-20 at 24 h. The genes of Gene Ontology terms with the highest statistical significance (<10−16) are listed and their presence in each term is annotated. (DOC) [file pone.0044870.s012.doc]

**Table S1.**

**Table S1 (continued).**
