# Supplementary material for: Multiparametric Analyses of Human PBMCs Loaded Ex Vivo with a Candidate Idiotype Vaccine for HCV-Related Lymphoproliferative Disorders
Source: PLoS One. 2012 Sep 18;7(9):e44870. doi: 10.1371/journal.pone.0044870 (PMC3445594; doi:10.1371/journal.pone.0044870)
Supplement: Table S3 — List of unique genes up-regulated by IGKV3-20 in PBMCs from HCV negative subjects at 24 h. (DOC) [file pone.0044870.s014.doc]

**Table S3.**

| **Gene ID** | **Gene symbol** | **Gene name** | **Gene ID** | **Gene symbol** | **Gene name** |
| --- | --- | --- | --- | --- | --- |
| 7958913 | OAS2 | 2’-5’-oligoadenylate synthetase 2, 69/71kDa | 7923907 | IL10 | interleukin 10 |
| 7958895 | OAS3 | 2’-5’-oligoadenylate synthetase 3, 100kDa | 8176323 | IL3RA | interleukin 3 receptor, alpha (low affinity) |
| 8103951 | ACSL1 | acyl-CoA synthetase long-chain family member 1 | 8095680 | IL8 | interleukin 8 |
| 7933772 | ANK3 | ankyrin 3, node of Ranvier (ankyrin G) | 8180377 | MXRA7 | matrix-remodelling associated 7 |
| 7983910 | AQP9 | aquaporin 9 | 7995829 | MT1H | metallothionein 1H |
| 7927732 | ARID5B | AT rich interactive domain 5B (MRF1-like) | 8068022 | MIR155HG | MIR155 host gene (non-protein coding) |
| 7943413 | BIRC3 | baculoviral IAP repeat-containing 3 | 7917283 | MCOLN2 | mucolipin 2 |
| 8092691 | BCL6 | B-cell CLL/lymphoma 6 | 8139433 | MYO1G | myosin IG |
| 8104601 | BASP1 | brain abundant, membrane attached signal protein 1 | 8068713 | MX1 | myxovirus (influenza virus) resistance 1, interferon-inducible protein p78 (mouse) |
| 8156228 | CTSL1 | cathepsin L1 | 8068697 | MX2 | myxovirus (influenza virus) resistance 2 (mouse) |
| 7909332 | CD55 | CD55 molecule, decay accelerating factor for complement (Cromer blood group) | 7930074 | NFKB2 | nuclear factor of kappa light polypeptide gene enhancer in B-cells 2 (p49/p100) |
| 7961075 | CD69 | CD69 molecule | 7978644 | NFKBIA | nuclear factor of kappa light polypeptide gene enhancer in B-cells inhibitor, alpha |
| 7939546 | CD82 | CD82 molecule | 7969288 | OLFM4 | olfactomedin 4 |
| 8006602 | CCL4 | chemokine (C-C motif) ligand 4 | 7934161 | PRF1 | perforin 1 (pore forming protein) |
| 8095697 | CXCL1 | chemokine (C-X-C motif) ligand 1 (melanoma growth stimulating activity, alpha) | 7923662 | PIK3C2B | phosphoinositide-3-kinase, class 2, beta polypeptide |
| 8100984 | CXCL3 | chemokine (C-X-C motif) ligand 3 | 8172471 | PIM2 | pim-2 oncogene |
| 8100977 | CXCL5 | chemokine (C-X-C motif) ligand 5 | 8101429 | PLAC8 | placenta-specific 8 |
| 7971486 | C13orf18 | chromosome 13 open reading frame 18 | 8065607 | PLAGL2 | pleiomorphic adenoma gene-like 2 |
| 7983478 | C15orf48 | chromosome 15 open reading frame 48 | 7934133 | PPA1 | pyrophosphatase (inorganic) 1 |
| 8025285 | C19orf59 | chromosome 19 open reading frame 59 | 8040080 | RSAD2 | radical S-adenosyl methionine domain containing 2 |
| 7956076 | CDK2 | cyclin-dependent kinase 2 | 8147206 | RIPK2 | receptor-interacting serine-threonine kinase 2 |
| 8047692 | CTLA4 | cytotoxic T-lymphocyte-associated protein 4 | 7979455 | RTN1 | reticulon 1 |
| 7946478 | DENND5A | DENN/MADD domain containing 5A | 8117106 | RNF144B | ring finger protein 144B |
| 7958019 | DRAM1 | DNA-damage regulated autophagy modulator 1 | 8066493 | SLPI | secretory leukocyte peptidase inhibitor |
| 7928308 | DDIT4 | DNA-damage-inducible transcript 4 | 7981068 | SERPINA1 | serpin peptidase inhibitor, clade A (alpha-1 antiproteinase, antitrypsin), member 1 |
| 8109999 | ERGIC1 | endoplasmic reticulum-golgi intermediate compartment (ERGIC) 1 | 8021635 | SERPINB2 | serpin peptidase inhibitor, clade B (ovalbumin), member 2 |
| 8095728 | EREG | epiregulin | 7921652 | SLAMF1 | signaling lymphocytic activation molecule family member 1 |
| 8136940 | FAM115C | family with sequence similarity 115, member C | 7906613 | SLAMF7 | SLAM family member 7 |
| 8081341 | FAM172B | family with sequence similarity 172, member B pseudogene | 8061227 | SLC24A3 | solute carrier family 24 (sodium/potassium/calcium exchanger), member 3 |
| 7904361 | FAM46C | family with sequence similarity 46, member C | 7983650 | SLC27A2 | solute carrier family 27 (fatty acid transporter), member 2 |
| 8167930 | FAAH2 | fatty acid amide hydrolase 2 | 7965964 | SLC41A2 | solute carrier family 41, member 2 |
| 7986092 | FURIN | furin (paired basic amino acid cleaving enzyme) | 8122986 | SNX9 | sorting nexin 9 |
| 7967318 | GPR109A | G protein-coupled receptor 109A | 8121794 | SMPDL3A | sphingomyelin phosphodiesterase, acid-like 3A |
| 7963770 | GPR84 | G protein-coupled receptor 84 | 8066038 | SLA2 | Src-like-adaptor 2 |
| 7996100 | GPR97 | G protein-coupled receptor 97 | 8020702 | TAF4B | TAF4b RNA polymerase II, TATA box binding protein (TBP)-associated factor, 105kDa |
| 7930894 | GRK5 | G protein-coupled receptor kinase 5 | 8131600 | TSPAN13 | tetraspanin 13 |
| 8096361 | HERC5 | hect domain and RLD 5 | 8163185 | TXN | thioredoxin |
| 8115455 | HAVCR1 | hepatitis A virus cellular receptor 1 | 7930537 | TCF7L2 | transcription factor 7-like 2 (T-cell specific, HMG-box) |
| 8116910 | HIVEP1 | human immunodeficiency virus type I enhancer binding protein 1 | 8126303 | TREM1 | triggering receptor expressed on myeloid cells 1 |
| 7937335 | IFITM1 | interferon induced transmembrane protein 1 (9-27) | 7897877 | TNFRSF1B | tumor necrosis factor receptor superfamily, member 1B |
| 8114010 | IRF1 | interferon regulatory factor 1 | 8074606 | USP18 | ubiquitin specific peptidase 18 |
| 7914127 | IFI6 | interferon, alpha-inducible protein 6 | 8088180 | WNT5A | wingless-type MMTV integration site family, member 5A |
| 7902553 | IFI44 | interferon-induced protein 44 | 8004184 | XAF1 | XIAP associated factor 1 |
| 7902541 | IFI44L | interferon-induced protein 44-like |  |  |  |
