# Supplementary material for: Multiparametric Analyses of Human PBMCs Loaded Ex Vivo with a Candidate Idiotype Vaccine for HCV-Related Lymphoproliferative Disorders
Source: PLoS One. 2012 Sep 18;7(9):e44870. doi: 10.1371/journal.pone.0044870 (PMC3445594; doi:10.1371/journal.pone.0044870)
Supplement: Table S4 — List of unique genes up-regulated by IGKV3-20 in PBMCs from HCV positive subjects at 24 h. (DOC) [file pone.0044870.s015.doc]

**Table S4.**

| **Gene ID** | **Gene symbol** | **Gene name** |
| --- | --- | --- |
| 8154733 | ACO1 | aconitase 1, soluble |
| 8115490 | ADAM19 | ADAM metallopeptidase domain 19 |
| 7985285 | ARNT2 | aryl-hydrocarbon receptor nuclear translocator 2 |
| 8162940 | ABCA1 | ATP-binding cassette, sub-family A (ABC1), member 1 |
| 8068761 | ABCG1 | ATP-binding cassette, sub-family G (WHITE), member 1 |
| 8010287 | C1QTNF1 | C1q and tumor necrosis factor related protein 1 |
| 7996022 | CCL22 | chemokine (C-C motif) ligand 22 |
| 8178115 | CFB | complement factor B |
| 7960900 | CLEC4E | C-type lectin domain family 4, member E |
| 7982854 | DLL4 | delta-like 4 (Drosophila) |
| 8117900 | DDR1 | discoidin domain receptor tyrosine kinase 1 |
| 8152522 | ENPP2 | ectonucleotide pyrophosphatase/phosphodiesterase 2 |
| 7939314 | EHF | ets homologous factor |
| 7985310 | FAM108C1 | family with sequence similarity 108, member C1 |
| 8136115 | FAM40B | family with sequence similarity 40, member B |
| 8098103 | FNIP2 | folliculin interacting protein 2 |
| 7970441 | GJB2 | gap junction protein, beta 2, 26kDa |
| 8151457 | HEY1 | hairy/enhancer-of-split related with YRPW motif 1 |
| 7919787 | HORMAD1 | HORMA domain containing 1 |
| 8031992 | FLJ45340 | hypothetical LOC402483 |
| 8146105 | IDO2 | indoleamine 2,3-dioxygenase 2 |
| 8044541 | IL1F9 | interleukin 1 family, member 9 |
| 7924029 | LAMB3 | laminin, beta 3 |
| 8070961 | LSS | lanosterol synthase (2,3-oxidosqualene-lanosterol cyclase) |
| 8031238 | LILRB4 | leukocyte immunoglobulin-like receptor, subfamily B (with TM and ITIM domains), member 4 |
| 8123176 | MAS1 | MAS1 oncogene |
| 8170420 | MAMLD1 | mastermind-like domain containing 1 |
| 7951271 | MMP1 | matrix metallopeptidase 1 (interstitial collagenase) |
| 8062023 | MAP1LC3A | microtubule-associated protein 1 light chain 3 alpha |
| 7939751 | NR1H3 | nuclear receptor subfamily 1, group H, member 3 |
| 8048257 | PNKD | paroxysmal nonkinesigenic dyskinesia |
| 8062927 | PI3 | peptidase inhibitor 3, skin-derived |
| 7919168 | PDE4DIP | phosphodiesterase 4D interacting protein |
| 8037775 | PTGIR | prostaglandin I2 (prostacyclin) receptor (IP) |
| 8149264 | PPP1R3B | protein phosphatase 1, regulatory (inhibitor) subunit 3B |
| 7972805 | RAB20 | RAB20, member RAS oncogene family |
| 7922717 | RGS16 | regulator of G-protein signaling 16 |
| 8021623 | SERPINB7 | serpin peptidase inhibitor, clade B (ovalbumin), member 7 |
| 8038877 | SIGLEC5 | sialic acid binding Ig-like lectin 5 |
| 7956759 | SRGAP1 | SLIT-ROBO Rho GTPase activating protein 1 |
| 8063382 | SNAI1 | snail homolog 1 (Drosophila) |
| 8003298 | SLC7A5 | solute carrier family 7 (cationic amino acid transporter, y+ system), member 5 |
| 8063923 | SLCO4A1 | solute carrier organic anion transporter family, member 4A1 |
| 8155707 | TJP2 | tight junction protein 2 (zona occludens 2) |
