# Supplementary material for: Multiparametric Analyses of Human PBMCs Loaded Ex Vivo with a Candidate Idiotype Vaccine for HCV-Related Lymphoproliferative Disorders
Source: PLoS One. 2012 Sep 18;7(9):e44870. doi: 10.1371/journal.pone.0044870 (PMC3445594; doi:10.1371/journal.pone.0044870)
Supplement: Table S5 — List of unique genes up-regulated by IGKV3-20 in PBMCs from HCV negative subjects at 6 d. (DOC) [file pone.0044870.s016.doc]

**Table S5.**

| **Gene ID** | **Gene symbol** | **Gene name** | **Gene ID** | **Gene symbol** | **Gene name** |
| --- | --- | --- | --- | --- | --- |
| 8146000 | ADAM9 | ADAM metallopeptidase domain 9 | 7956878 | IRAK3 | interleukin-1 receptor-associated kinase 3 |
| 8092970 | APOD | apolipoprotein D | 7916609 | JUN | jun proto-oncogene |
| 7927215 | ALOX5 | arachidonate 5-lipoxygenase | 8035456 | LRRC25 | leucine rich repeat containing 25 |
| 8145454 | BNIP3L | BCL2/adenovirus E1B 19kDa interacting protein 3-like | 7954810 | LRRK2 | leucine-rich repeat kinase 2 |
| 8081171 | CRYBG3 | beta-gamma crystallin domain containing 3 | 7940259 | MS4A7 | membrane-spanning 4-domains, subfamily A, member 7 |
| 7961829 | BCAT1 | branched chain amino-acid transaminase 1, cytosolic | 8163775 | MEGF9 | multiple EGF-like-domains 9 |
| 8068105 | BACH1 | BTB and CNC homology 1, basic leucine zipper transcription factor 1 | 8047127 | MYO1B | myosin IB |
| 8057578 | CALCRL | calcitonin receptor-like | 7911178 | NLRP3 | NLR family, pyrin domain containing 3 |
| 8006123 | CPD | carboxypeptidase D | 8068100 | NCRNA00189 | non-protein coding RNA 189 |
| 8114612 | CD14 | CD14 molecule | 8140468 | PION | pigeon homolog (Drosophila) |
| 8056102 | CD302 | CD302 molecule | 7898057 | PDPN | podoplanin |
| 8079392 | CCR2 | chemokine (C-C motif) receptor 2 | 8074714 | POM121L1P | POM121 membrane glycoprotein-like 1, pseudogene |
| 8011713 | CXCL16 | chemokine (C-X-C motif) ligand 16 | 8020110 | RAB31 | RAB31, member RAS oncogene family |
| 7968883 | C13orf31 | chromosome 13 open reading frame 31 | 8013753 | RAB34 | RAB34, member RAS oncogene family |
| 8160531 | C9orf72 | chromosome 9 open reading frame 72 | 7972946 | RASA3 | RAS p21 protein activator 3 |
| 8044391 | MERTK | c-mer proto-oncogene tyrosine kinase | 7977003 | RCOR1 | REST corepressor 1 |
| 8176306 | CSF2RA | colony stimulating factor 2 receptor, alpha, low-affinity (granulocyte-macrophage) | 8108873 | ARHGAP26 | Rho GTPase activating protein 26 |
| 8172333 | CFP | complement factor properdin | 8122637 | SASH1 | SAM and SH3 domain containing 1 |
| 8046333 | CYBRD1 | cytochrome b reductase 1 | 8069541 | SAMSN1 | SAM domain, SH3 domain and nuclear localization signals 1 |
| 8171105 | CRLF2 | cytokine receptor-like factor 2 | 7946516 | SBF2 | SET binding factor 2 |
| 8145365 | DOCK5 | dedicator of cytokinesis 5 | 7925062 | SIPA1L2 | signal-induced proliferation-associated 1 like 2 |
| 8022711 | DSC2 | desmocollin 2 | 8157038 | SLC44A1 | solute carrier family 44, member 1 |
| 8115831 | DUSP1 | dual specificity phosphatase 1 | 8068361 | SLC5A3 | solute carrier family 5 (sodium/myo-inositol cotransporter), member 3 |
| 8034851 | EMR3 | egf-like module containing, mucin-like, hormone receptor-like 3 | 8102800 | SLC7A11 | solute carrier family 7, (cationic amino acid transporter, y+ system) member 11 |
| 8022118 | EPB41L3 | erythrocyte membrane protein band 4.1-like 3 | 8060745 | SMOX | spermine oxidase |
| 8017867 | FAM20A | family with sequence similarity 20, member A | 7993624 | SYT17 | synaptotagmin XVII |
| 8050427 | FAM49A | family with sequence similarity 49, member A | 8180319 | TGIF1 | TGFB-induced factor homeobox 1 |
| 7975779 | FOS | FBJ murine osteosarcoma viral oncogene homolog | 7982597 | THBS1 | thrombospondin 1 |
| 8106141 | FCHO2 | FCH domain only 2 | 8136557 | TBXAS1 | thromboxane A synthase 1 (platelet) |
| 8179688 | FLOT1 | flotillin 1 | 8001818 | TK2 | thymidine kinase 2, mitochondrial |
| 8038899 | FPR1 | formyl peptide receptor 1 | 8052872 | TGFA | transforming growth factor, alpha |
| 7910680 | GPR137B | G protein-coupled receptor 137B | 8152453 | TRPS1 | trichorhinophalangeal syndrome I |
| 8072926 | H1F0 | H1 histone family, member 0 | 7983734 | TMOD2 | tropomodulin 2 (neuronal) |
| 8005097 | HS3ST3B1 | heparan sulfate (glucosamine) 3-O-sulfotransferase 3B1 | 8174361 | TSC22D3 | TSC22 domain family, member 3 |
| 7974851 | HIF1A | hypoxia inducible factor 1, alpha subunit (basic helix-loop-helix transcription factor) | 8129637 | VNN2 | vanin 2 |
| 8044766 | INSIG2 | insulin induced gene 2 | 8066266 | MAFB | v-maf musculoaponeurotic fibrosarcoma oncogene homolog B (avian) |
| 8046861 | ITGAV | integrin, alpha V (vitronectin receptor, alpha polypeptide, antigen CD51) | 8146500 | LYN | v-yes-1 Yamaguchi sarcoma viral related oncogene homolog |
| 8169580 | IL13RA1 | interleukin 13 receptor, alpha 1 | 8098470 | WWC2 | WW and C2 domain containing 2 |
