# Supplementary material for: Multiparametric Analyses of Human PBMCs Loaded Ex Vivo with a Candidate Idiotype Vaccine for HCV-Related Lymphoproliferative Disorders
Source: PLoS One. 2012 Sep 18;7(9):e44870. doi: 10.1371/journal.pone.0044870 (PMC3445594; doi:10.1371/journal.pone.0044870)
Supplement: Table S6 — List of unique genes up-regulated by IGKV3-20 in PBMCs from HCV positive subjects at 6 d. (DOC) [file pone.0044870.s017.doc]

**Table S6.**

| **Gene ID** | **Gene symbol** | **Gene name** | **Gene ID** | **Gene symbol** | **Gene name** |
| --- | --- | --- | --- | --- | --- |
| 7990810 | MTHFS | 5,10-methenyltetrahydrofolate synthetase (5-formyltetrahydrofolate cyclo-ligase) | 7983910 | AQP9 | aquaporin 9 |
| 7990815 | ST20 | suppressor of tumorigenicity 20 | 7959834 | GLT1D1 | glycosyltransferase 1 domain containing 1 |
| 7968556 | KL | klotho | 8102594 | TNIP3 | TNFAIP3 interacting protein 3 |
| 8034851 | EMR3 | egf-like module containing, mucin-like, hormone receptor-like 3 | 8038899 | FPR1 | formyl peptide receptor 1 |
| 8157038 | SLC44A1 | solute carrier family 44, member 1 | 8068583 | KCNJ15 | potassium inwardly-rectifying channel, subfamily J, member 15 |
| 8013753 | RAB34 | RAB34, member RAS oncogene family | 8180196 | LILRA6 | leukocyte immunoglobulin-like receptor, subfamily A (with TM domain), member 6 |
| 8087935 | NT5DC2 | 5’-nucleotidase domain containing 2 | 7921882 | OLFML2B | olfactomedin-like 2B |
| 8018864 | SOCS3 | suppressor of cytokine signaling 3 | 8017867 | FAM20A | family with sequence similarity 20, member A |
| 8132725 | UPP1 | uridine phosphorylase 1 | 7975459 | SIPA1L1 | signal-induced proliferation-associated 1 like 1 |
| 7962689 | VDR | vitamin D (1,25- dihydroxyvitamin D3) receptor | 7986092 | FURIN | furin (paired basic amino acid cleaving enzyme) |
| 8117334 | HIST1H4A | histone cluster 1, H4a | 8166632 | GK | glycerol kinase |
| 8117543 | HIST1H2AH | histone cluster 1, H2ah | 7981068 | SERPINA1 | serpin peptidase inhibitor, clade A (alpha-1 antiproteinase, antitrypsin), member 1 |
| 8124540 | HIST1H2AM | histone cluster 1, H2am | 8119898 | VEGFA | vascular endothelial growth factor A |
| 8117583 | HIST1H2AI | histone cluster 1, H2ai | 8031223 | LILRB1 | leukocyte Ig-like receptor, subfamily B (with TM and ITIM domains), member 1 |
| 8124397 | HIST1H1C | histone cluster 1, H1c | 8039166 | MBOAT7 | membrane bound O-acyltransferase domain containing 7 |
| 7956076 | CDK2 | cyclin-dependent kinase 2 | 8171105 | CRLF2 | cytokine receptor-like factor 2 |
| 7919614 | HIST2H3A | histone cluster 2, H3a | 8039212 | LILRB2 | leukocyte Ig-like receptor, subfamily B (with TM and ITIM domains), member 2 |
| 8124537 | HIST1H3J | histone cluster 1, H3j | 8011713 | CXCL16 | chemokine (C-X-C motif) ligand 16 |
| 8015769 | BRCA1 | breast cancer 1, early onset | 8163775 | MEGF9 | multiple EGF-like-domains 9 |
| 8117608 | HIST1H2AL | histone cluster 1, H2al | 8167185 | TIMP1 | TIMP metallopeptidase inhibitor 1 |
| 8092691 | BCL6 | B-cell CLL/lymphoma 6 | 8173287 | VSIG4 | V-set and Ig domain containing 4 |
| 7922416 | SNORD75 | small nucleolar RNA, C/D box 75 | 7974851 | HIF1A | hypoxia inducible factor 1, alpha subunit (basic helix-loop-helix transcription factor) |
| 8013965 | SSH2 | slingshot homolog 2 (Drosophila) | 8006123 | CPD | carboxypeptidase D |
| 7932407 | ST8SIA6 | ST8 alpha-N-acetyl-neuraminide alpha-2,8-sialyltransferase 6 | 7914878 | CLSPN | claspin |
| 8124521 | HIST1H4K | histone cluster 1, H4k | 8120210 | IL17A | interleukin 17A |
| 7920244 | S100A8 | S100 calcium binding protein A8 | 7983650 | SLC27A2 | solute carrier family 27 (fatty acid transporter), member 2 |
| 8102800 | SLC7A11 | solute carrier family 7, (cationic a. a. transporter, y+ system) member 11 | 7986068 | BLM | Bloom syndrome, RecQ helicase-like |
| 7898057 | PDPN | podoplanin | 7962183 | AK4 | adenylate kinase 4 |
| 7905571 | S100A9 | S100 calcium binding protein A9 | 8124413 | HIST1H4D | histone cluster 1, H4d |
| 7989501 | CA12 | carbonic anhydrase XII | 7993624 | SYT17 | synaptotagmin XVII |
| 8095886 | CXCL13 | chemokine (C-X-C motif) ligand 13 | 8005097 | HS3ST3B1 | heparan sulfate (glucosamine) 3-O-sulfotransferase 3B1 |
| 8018189 | CD300E | CD300e molecule | 8174103 | GK | glycerol kinase |
| 7939546 | CD82 | CD82 molecule | 8068100 | NCRNA00189 | non-protein coding RNA 189 |
| 8135601 | MET | met proto-oncogene (hepatocyte growth factor receptor) | 8072461 | LIMK2 | LIM domain kinase 2 |
| 8131666 | ITGB8 | integrin, beta 8 | 7995838 | MT1X | metallothionein 1X |
| 8048283 | SLC11A1 | solute carrier family 11 (proton-coupled divalent metal ion transp), member 1 | 7951385 | CASP5 | caspase 5, apoptosis-related cysteine peptidase |
| 8106743 | VCAN | versican | 8146092 | IDO1 | indoleamine 2,3-dioxygenase 1 |
| 8128939 | TRAF3IP2 | TRAF3 interacting protein 2 | 7927732 | ARID5B | AT rich interactive domain 5B (MRF1-like) |
| 8129637 | VNN2 | vanin 2 | 8019954 | FLJ35776 | hypothetical LOC649446 |
| 8030860 | FPR2 | formyl peptide receptor 2 | 8139433 | MYO1G | myosin IG |
| 7979131 | GNG2 | guanine nucleotide binding protein (G protein), gamma 2 | 7921868 | FCGR3A | Fc fragment of IgG, low affinity IIIa, receptor (CD16a) |
| 7953749 | CLEC4D | C-type lectin domain family4, member D | 8079392 | CCR2 | chemokine (C-C motif) receptor 2 |
| 8025103 | EMR1 | egf-like module containing, mucin-like, hormone receptor-like 1 | 8072170 | KREMEN1 | kringle containing transmembrane protein 1 |
| 7982597 | THBS1 | thrombospondin 1 |  |  |  |
